# Aberrant Expression of A Disintegrin and Metalloproteinase With Thrombospondin Motifs 13 (ADAMTS13) in Pancreatic Cancer Leads to Dichotomic Functions

**DOI:** 10.1002/mco2.70462

**Published:** 2025-11-06

**Authors:** Stephanie Allmang, Hagen R. Witzel, Anne Hausen, Simone Marquard, Christoph Eckert, Nicole Marnet, Nina Hörner, Philipp Mayer, Stefan Heinrich, Hien Dang, Wilfried Roth, Matthias M. Gaida

**Affiliations:** ^1^ Institute of Pathology University Medical Center Mainz Mainz Germany; ^2^ TRON Translational Oncology at the University Medical Center Mainz Mainz Germany; ^3^ Clinic For Diagnostic and Interventional Radiology University Hospital Heidelberg Heidelberg Germany; ^4^ Department of Surgery University Medical Center Mainz Mainz Germany; ^5^ Department of Surgery Medical Campus Trier Mutterhaus Klinik Trier Germany; ^6^ Department of Surgery Department of Surgical Research Thomas Jefferson University Philadelphia Pennsylvania USA; ^7^ Research Center for Immunotherapy University Medical Center Mainz Mainz Germany

**Keywords:** ADAMTS13, adipokines, pancreas, PDAC, peripancreatic adipose tissue, vascularization

## Abstract

Pancreatic ductal adenocarcinoma (PDAC) is one of the most aggressive cancers characterized by highly invasive growth into the surrounding peripancreatic fat tissue, where tumor cells can directly interact with adipocytes. Due to poor response to the currently available (radio)chemotherapies, there is an urgent need for advanced therapy concepts. The present study shows that ADAMTS13 (a disintegrin and metalloproteinase with thrombospondin motifs 13), a key factor in blood coagulation, is significantly overexpressed in human PDAC. Immunohistochemical analysis revealed that ADAMTS13 expression is associated with prolonged survival and negatively correlated with vascular density. In vitro and in vivo experiments demonstrate its partial induction by leptin. Mechanistically, CRISPR/Cas‐mediated *ADAMTS13* knockout in PDAC cells resulted in reduced migration and invasion. In an avian xenograft tumor model, ADAMTS13 loss led to increased vascularization, decreased vascular length, and diminished tumor growth, accompanied by reduced expression of multiple key angiogenic and angioplastic factors. Furthermore, loss of ADAMTS13 was associated with decreased expression of mesenchymal markers. In conclusion, we identified an aberrant expression and alternative function of ADAMTS13 in PDAC linked to tumor progression, plasticity, and angiogenesis, partly induced by the peripancreatic fat tissue, making this metalloproteinase an interesting target for personalized therapies.

## Graphical abstract

Allmang et al. demonstrated that ADAMTS13 is overexpressed in PDAC, associated with prolonged survival and reduced vascular density. Knockout of *ADAMTS13* in PDAC cells led to a decrease in migration and invasion. In a xenograft model, loss of ADAMTS13 altered angiogenesis, vascular maturation, and reduced the expression of key angiogenic factors, highlighting its potential as a therapeutic target.

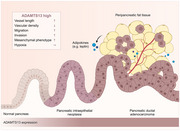

## 1 Introduction

Pancreatic ductal adenocarcinoma (PDAC) is among the most aggressive cancer types, with a 5‐year survival rate of less than 13% [1]. Projections indicate that by 2030, PDAC will emerge as the second leading cause of cancer‐related mortality [2]. PDAC is usually diagnosed at advanced stages, has limited treatment options, and a notoriously poor response to currently available (radio)chemotherapies. Notably, only ∼20% of patients are eligible for potentially curative surgery highlighting the urgent need for individualized therapy concepts [3, 4]. PDAC progression is driven by an infiltrative tumor growth, rapid vascular invasion, and early metastatic spread. Locally, tumor cells at the invasive margin frequently infiltrate the peripancreatic adipose tissue, an anatomical layer covering the pancreas and consisting mainly of adipocytes [5]. Adipocytes secrete a variety of different adipokines with multiple humoral and local paracrine effects [6]. It is documented that elevated serum levels of the adipokine leptin are associated with an increased PDAC risk [7]. Moreover, leptin can promote cancer cell proliferation and their glucose/lactate metabolism [8].

Proteolytic enzymes have also been implicated as key mediators in PDAC progression, particularly through the cleavage of adhesion molecules, which facilitates tumor cell detachment, migration, invasion, and neoangiogenesis [9, 10]. Among these, members of the ADAMTS (a disintegrin and metalloproteinase with thrombospondin motifs) family of secreted metalloproteinases have emerged as key modulators of extracellular matrix remodeling, inflammation, and vascular biology driving tissue morphogenesis and physiological or pathological remodeling [11, 12]. This heterogeneous protein family comprises 19 members, each with distinct but sometimes overlapping functions [12]. ADAMTS13, a unique member of the protease family, regulates blood coagulation by cleaving von Willebrand factor (VWF) multimers, thereby preventing spontaneous platelet aggregation. Impaired ADAMTS13 activity has been linked to an increased risk of venous thromboembolism (VTE), a potentially life‐threatening complication frequently observed during the progression of pancreatic, gastric, and cerebral cancers [13, 14].

Recent studies have already implicated ADAMTS family members in PDAC, with ADAMTS12 promoting cell migration, modulating epithelial‐to‐mesenchymal transition (EMT), and correlating with poor prognosis [15]. The analysis of a small PDAC cohort showed that ADAMTS1, 8, 9, and 18 were upregulated in tumor tissue compared to non‐neoplastic adjacent tissue, which was associated with lymph node metastases [16]. Together, these data provide strong evidence implicating ADAMTS proteases as important drivers of PDAC progression. However, ADAMTS13's role in tumor progression remains largely unknown.

This study aimed to evaluate the role of ADAMTS13 in PDAC, revealing its elevated expression and association with prolonged patient survival. Its depletion reduced migration and invasion in vitro and led to increased vascularization, shorter vessel length, and reduced tumor growth in vivo. This was accompanied by decreased expression of angiogenic and mesenchymal markers. These findings highlight the potential of this metalloproteinase as an intriguing target for personalized therapies in PDAC.

## 2 RESULTS

### 2.1 ADAMTS13 Expression Profiles During Carcinogenesis of PDAC

To investigate ADAMTS13 expression in PDAC tissue, we conducted immunohistochemical analysis of a tissue microarray (TMA) comprising samples from 112 patients, including non‐neoplastic pancreatic tissue, low and high‐grade pancreatic intraepithelial neoplasia (PanIN), PDAC tissue from the center and periphery, and lymph node metastases. Additionally, to validate the TMA and ensure staining consistency, we stained for matrixmetalloproteinase 12 (MMP12) and serine protease inhibitor 12 (SERPINA12), a protease known to be a prognostic marker of PDAC and a protease inhibitor, respectively [17].

**FIGURE 1:**
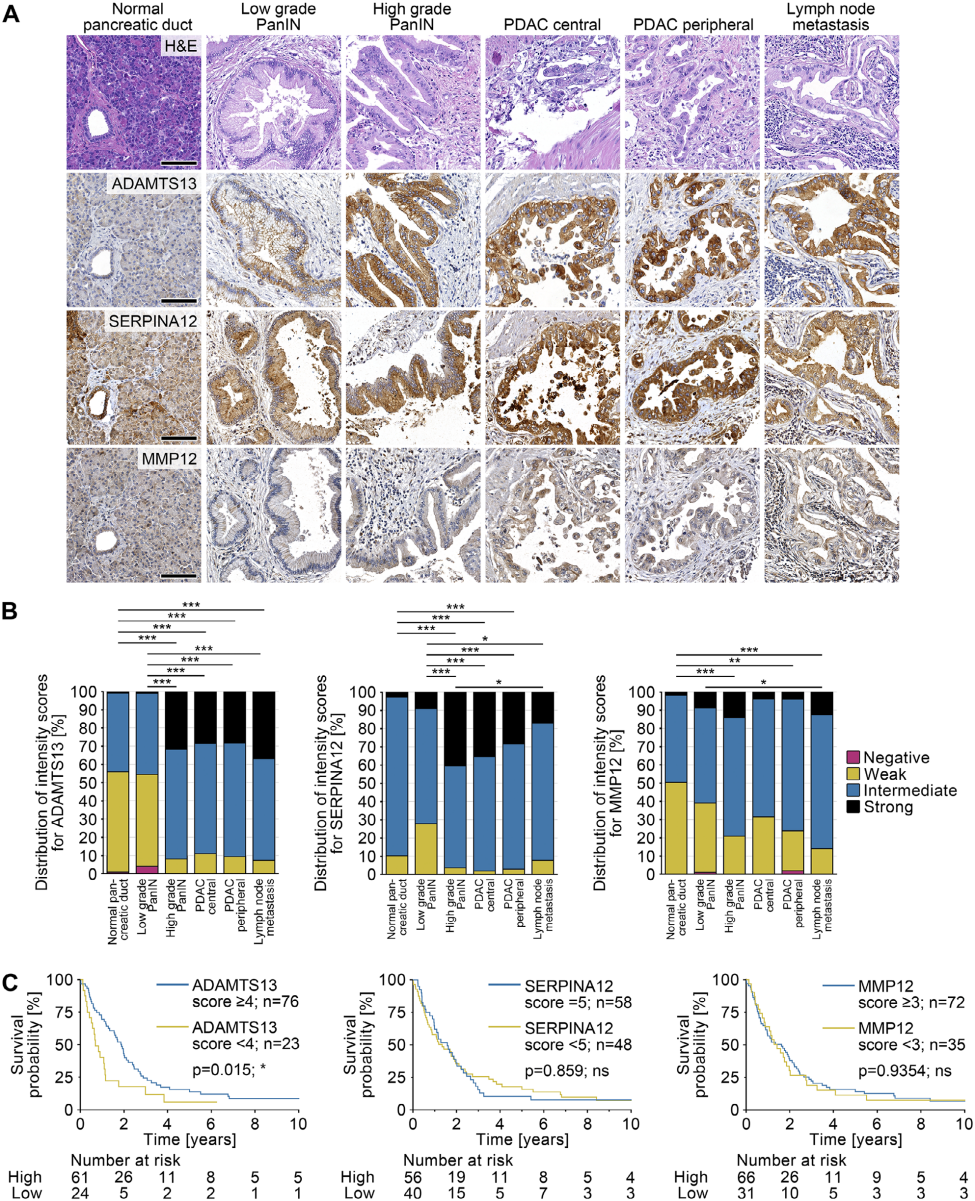
Expression profiles of ADAMTS13 during the development and progression of PDAC. (A) H&E and immunohistochemical analysis of ADAMTS13, SERPINA12, and MMP12 of a TMA. The TMA includes non‐neoplastic pancreatic tissue, low and high‐grade PanIN tissue, PDAC tissue from the center and periphery of the tumor, and, if available, tissue from lymph node metastases from a total of 112 patients. Scale bars 100 µm. (B) Quantification of the intensity of stained cells using the Allred intensity score (0‐3). The percentage distribution of the different intensities is shown. Kruskal‐Wallis test. (C) Comparison of Kaplan‐Meier survival curves between patients with high and low ADAMTS13 (≥ 4; *n* = 76 vs. < 4; *n* = 23), SERPINA12 (5; *n* = 58 vs. < 5; *n* = 48), and MMP12 (≥ 3; *n* = 72 vs. < 3; *n* = 35) expression. For the analysis, the intensity of the staining was again evaluated using a more sensitive scoring system (0–5). Log‐rank test. *p*‐values ≤ 0.05 (*), *p* ≤ 0.01 (**), *p* ≤ 0.001 were considered significant. *p‐*values > 0.05 were considered not significant.

In non‐neoplastic tissue as well as in non‐invasive precursor lesion low‐grade PanIN, the epithelial cells of the pancreatic ducts showed an overall weak‐positive and focal staining for ADAMTS13. Notably, the staining intensity of ADAMTS13 increased throughout PDAC progression with significantly higher expression in high‐grade PanIN (*p* < 0.001), invasive PDAC (central (*p* < 0.001), peripheral (*p* < 0.001)), and lymph node metastases (*p* < 0.001). While in low‐grade PanIN, the ADAMTS13 staining was restricted to the basal compartment of epithelial cells with papillary or micropapillary architecture, in high‐grade PanIN, ADAMTS13 was more ubiquitously localized within the cellular compartments. The staining intensity of PDAC cells was significantly stronger than in the precursor lesions (*p* < 0.001) and homogeneously distributed throughout the cell (Figures 1A,B and S1–S3).

The ductal epithelium of the non‐neoplastic tissue, as well as the low and high‐grade PanIN tissue, showed intermediate staining for SERPINA12, which was homogeneously localized in the cancer cells. SERPINA12 staining was not significantly increased in low‐grade PanIN compared to ductal epithelium of non‐neoplastic tissue (*p* > 0.99), whereas its expression was significantly increased in high‐grade PanIN tissue (*p* < 0.001). The expression of SERPINA12 in the central (*p* < 0.001) as well as peripheral tumor compartments (*p* < 0.001) showed a significant increase in expression compared to non‐neoplastic tissue. However, when compared to high‐grade PanIN, SERPINA12 staining was not significantly different (central: *p* > 0.99; peripheral: *p* > 0.99) (Figures 1A,B and S3).

MMP12 showed significantly lower staining intensity compared to ADAMTS13 (*p* < 0.001) and SERPINA12 (*p* < 0.001). The ductal epithelium of the non‐neoplastic tissue showed weak expression, low‐grade PanIN, and high‐grade PanIN tissue had a weak to intermediate expression. The staining intensity of the non‐neoplastic pancreatic tissue was significantly lower compared to high‐grade PanIN tissue (*p* < 0.001), peripheral parts of the tumor (*p* = 0.003), and lymph node metastases (*p* < 0.001). The staining intensities of the low‐grade PanIN were not significantly different from those of the central (*p* > 0.99) or peripheral parts of the tumor (*p* > 0.99) but significantly lower than in lymph node metastases (*p* = 0.02). Compared to the high expression in high‐grade PanIN, this was even significantly reduced in PDAC tissue (*p* = 0.006). No significant differences in the expression of ADAMTS13 (*p* > 0.99), SERPINA12 (*p* > 0.99), and MMP12 (*p* > 0.99) were found within the tumor (center vs. periphery; Figures 1A,B and S3).

Patients with a low ADAMTS13 expression (intensity score of < 4) showed a significantly shorter median survival (252 days vs. 690 days; *p* = 0.015) than patients with a high ADAMTS13 expression (intensity score of ≥ 4; Figure 1C). This association between high ADAMTS13 expression and prolonged survival was confirmed in publicly available data from the TCGA dataset (*p* = 0.0086) as well as in an independent cohort of patients who received neoadjuvant chemotherapy (*p* = 0.0445; Figures S4 and S5). Notably, the parameters of the tumor, nodes, metastases (TNM) classification, histological grading, age, and sex were not associated with ADAMTS13 expression. By contrast, SERPINA12 and MMP12 expression was not significantly associated with patient survival and did not reveal any correlation with clinical or pathological parameters either.

In summary, ADAMTS13 is markedly expressed at higher levels in human PDAC compared to non‐neoplastic tissues or low‐grade precursor lesions. Its expression is associated with prolonged survival. However, despite the similar induction of SERPINA12 and MMP12 expression in the tumor, this does not correlate with survival.

### 2.2 Differential Expression of ADAMTS13 in PDAC Tissue

**FIGURE 2:**
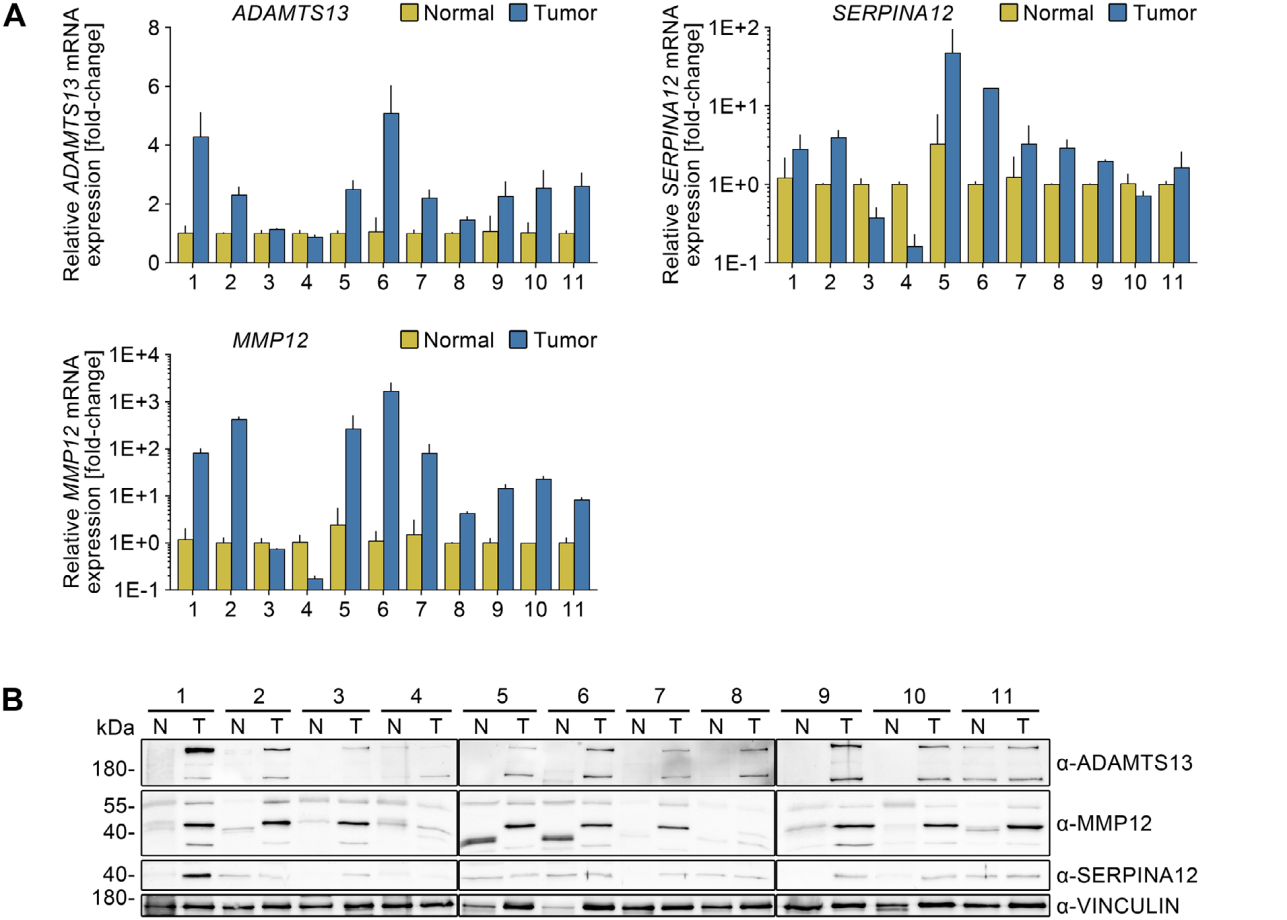
Strong differential expression of *ADAMTS13, SERPINA12*, and *MMP12* in PDAC tissue. (A) Semiquantitative real‐time PCR analysis for the proteases *ADAMTS13* and *MMP12* and the protease inhibitor *SERPINA12* in a collective of 11 PDAC (tumor, blue) and the respective adjacent non‐neoplastic tissues (normal, yellow). Each bar represents the mean and the respective standard deviation of two technical replicates. Expression was normalized to *RNA18S*. Relative expression in the respective normal tissues was set to 1. (B) Immunoblot analysis for ADAMTS13, MMP12, and SERPINA12 in the same collective. Tumor, T; respective adjacent non‐neoplastic tissues, N. Vinculin served as loading control. Molecular weight in kDa.

To validate the regulated factors at both the protein and mRNA level, an independent collection of 11 different patient tissues with a PDAC diagnosis was analyzed (Figures 2A,B, S6, and S7).

*ADAMTS13* (*p* = 0.002), *SERPINA12* (*p* = 0.0186), and *MMP12* (*p* = 0.0049) showed significantly increased expression at the mRNA level in the majority of tumor tissues compared to non‐neoplastic tissue (Figure 2A). Consistent with the mRNA expression data, analysis of the same samples at the protein level also showed elevated expression of ADAMTS13 and, to a lesser extent, MMP12 in tumor tissue (Figures 2B and S7). SERPINA12, on the other hand, was upregulated in only about half of the samples compared to the reference tissue, while no difference was observed in the remaining cases. Additional analyses of proteases and protease inhibitors revealed variable expression patterns across samples (Figure S6).

Accordingly, *ADAMTS13* and *MMP12* demonstrate robust expression at both the mRNA and protein levels in tumor tissue when compared to matching non‐neoplastic tissue.

### 2.3 Identification of Adipocyte‐Released Factors in PDAC

**FIGURE 3:**
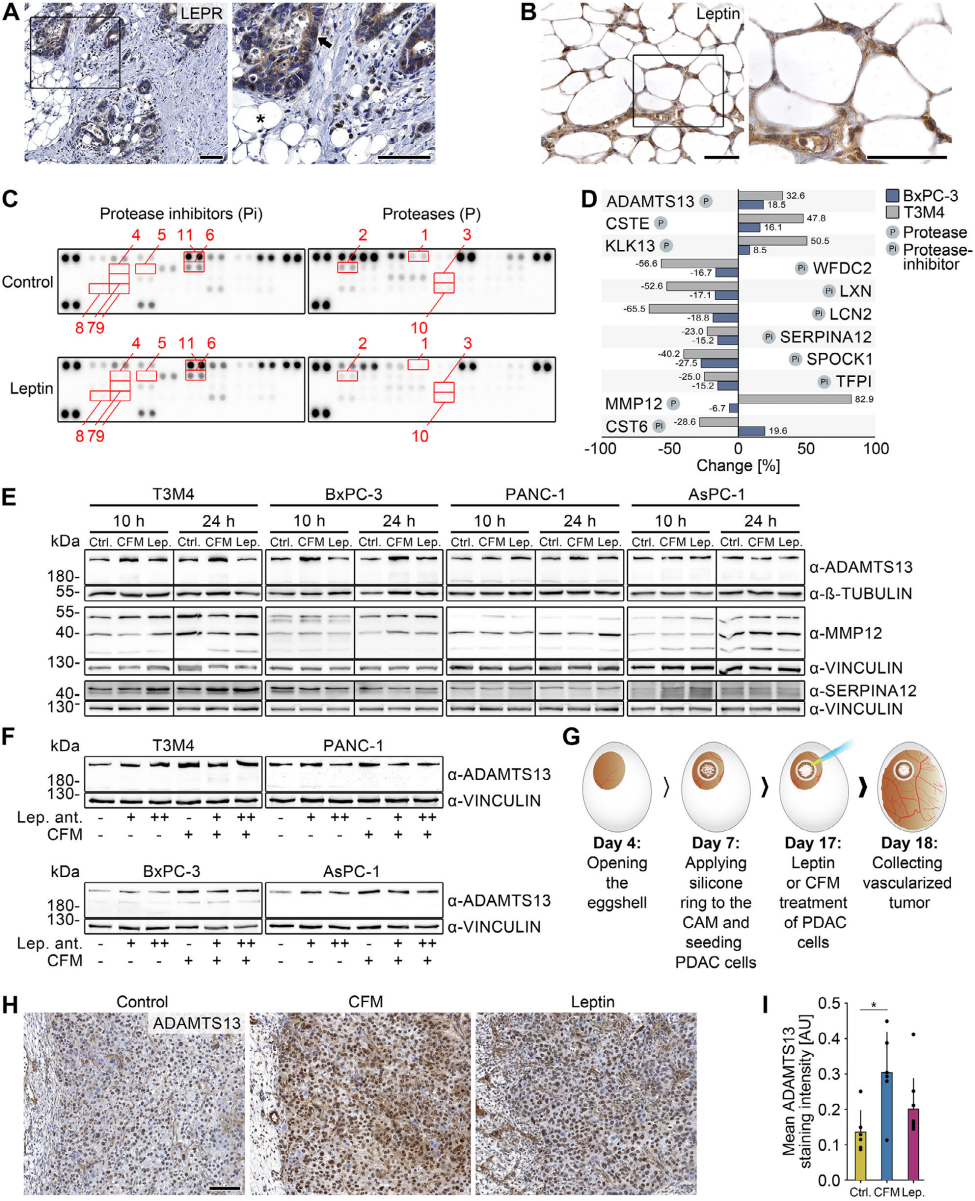
Identification of leptin‐regulated factors in PDAC. (A) Immunohistochemical analysis of the leptin receptor (LEPR) in PDAC and peripancreatic adipose tissue (PAT). PAT is indicated with an asterisk and cancer tissue with an arrow. Scale bars 100 µm. (B) Immunohistochemical analysis of leptin in PDAC and PAT. Scale bars 100 µm. (C, D) Immunoblot analysis of proteome profiler array for protease inhibitors (Pi) and proteases (P). BxPC‐3 or T3M4 cells were either treated with 10 ng/mL leptin or control medium for 24 h. Differentially expressed proteins are highlighted with red rectangles and listed in (D). The upper part of the table includes targets that showed the same trend in both cell lines, and the lower part of the table includes targets that were strongly but differently regulated in both cell lines. (E) Immunoblot analysis of ADAMTS13, MMP12, and SERPINA12 in the PDAC cell lines T3M4, BxPC‐3, PANC‐1, and AsPC‐1 treated with leptin (Lep.), conditioned fat medium (CFM) or control medium (Ctrl.) for 10 or 24 h. Vinculin and ß‐tubulin served as loading controls. Molecular weight in kDa. (F) Immunoblot analysis of ADAMTS13 in the PDAC cell lines T3M4, BxPC‐3, PANC‐1, and AsPC‐1 treated with CFM alone or in combination with 100 or 500 ng/mL leptin antagonist or control medium (Ctrl.) for 24 h. Vinculin served as loading control. Molecular weight in kDa. (G) Workflow of the chorioallantoic membrane (CAM) model. The applied cells proliferate, grow on the CAM, and form tumors that are infiltrated by CAM vessels over time. (H) Immunohistochemical staining for ADAMTS13 of T3M4 CAM xenograft tumors grown for 11 days and treated with leptin (*n* = 9), CFM (*n* = 6), or control medium (*n* = 6) for 1 day. Scale bar 100 µm. (I) Quantification of the ADAMTS13 staining intensity. Arbitrary unit (AU). Kruskal‐Wallis test. *p*‐values ≤ 0.05 (*) were considered significant. *p‐*values > 0.05 were considered not significant.

A frequent event in PDAC is the diffuse invasion of tumor cells into surrounding peripancreatic fat tissue. Our analyses revealed distinct patterns of adipocyte distribution, with tumor cells in either direct contact or separated by stroma (Figure S1). Cancer cell clusters were found in the peripancreatic adipose tissue and at the invasive margin, suggesting potential interaction with adipocytes. Intratumoral adipocytes were rare and appeared in small clusters, either adjacent to tumor cells or embedded in stroma (Figure S8). The close spatial proximity between adipocytes and neoplastic cells creates a direct interface for tumor–adipocyte crosstalk, suggesting that adipocyte‐derived factors may modulate PDAC cell behavior and contribute to disease progression. Among these factors, the adipokine leptin is of particular interest, as its receptor (LEPR) is expressed by PDAC cells (Figure 3A). In line with this, leptin expression was also detected in peripancreatic adipose tissue from PDAC patients (Figure 3B).

To investigate whether ADAMTS13 is regulated by leptin and to identify other leptin‐regulated targets, proteome profiler arrays covering a broad range of proteases and protease inhibitors were performed on lysates of leptin‐exposed tumor cells (Figure 3C). Stimulation of two PDAC cell lines (BxPC‐3 and T3M4) with leptin led to deregulation of different proteins, including ADAMTS13 and SERPINA12 (Figure 3D). The results of the screening were validated by semiquantitative real‐time PCR (Figure S9) and immunoblot analyses (Figures 3E and S10). Conditioned fat medium (CFM) as supernatant of cultivated human adipose tissue and thus a mixture of different adipokines and growth factors had significantly stronger effects than leptin alone, as visible for ADAMTS13 expression (Figure 3E). Consequently, treatment with a leptin antagonist diminished the induction of ADAMTS13 by CFM (Figures 3F and S11).

To examine the effects of adipokines, particularly leptin, on ADAMTS13 regulation during tumor development, we employed the chorioallantoic membrane (CAM) xenograft model (Figure 3G) [18]. Leptin treatment of T3M4 xenograft tumors led to a slight but non‐significant increase in ADAMTS13 expression (*p* = 0.37), whereas treatment with CFM significantly elevated its expression (*p* = 0.01; Figure 3H,I).

In conclusion, the secretion of factors, such as leptin, by the peripancreatic adipose tissue results in the stimulation of ADAMTS13 expression within cellular and xenograft models.

### 2.4 ADAMTS13 Mediates Migration and Invasion in PDAC

Early metastasis and chemoresistance of tumor cells represent hallmarks of PDAC progression. To investigate the influence of ADAMTS13 on these parameters, we employed CRISPR/Cas to genomically target *ADAMTS13* in T3M4 and PANC‐1 cells (Figure S12).

**FIGURE 4:**
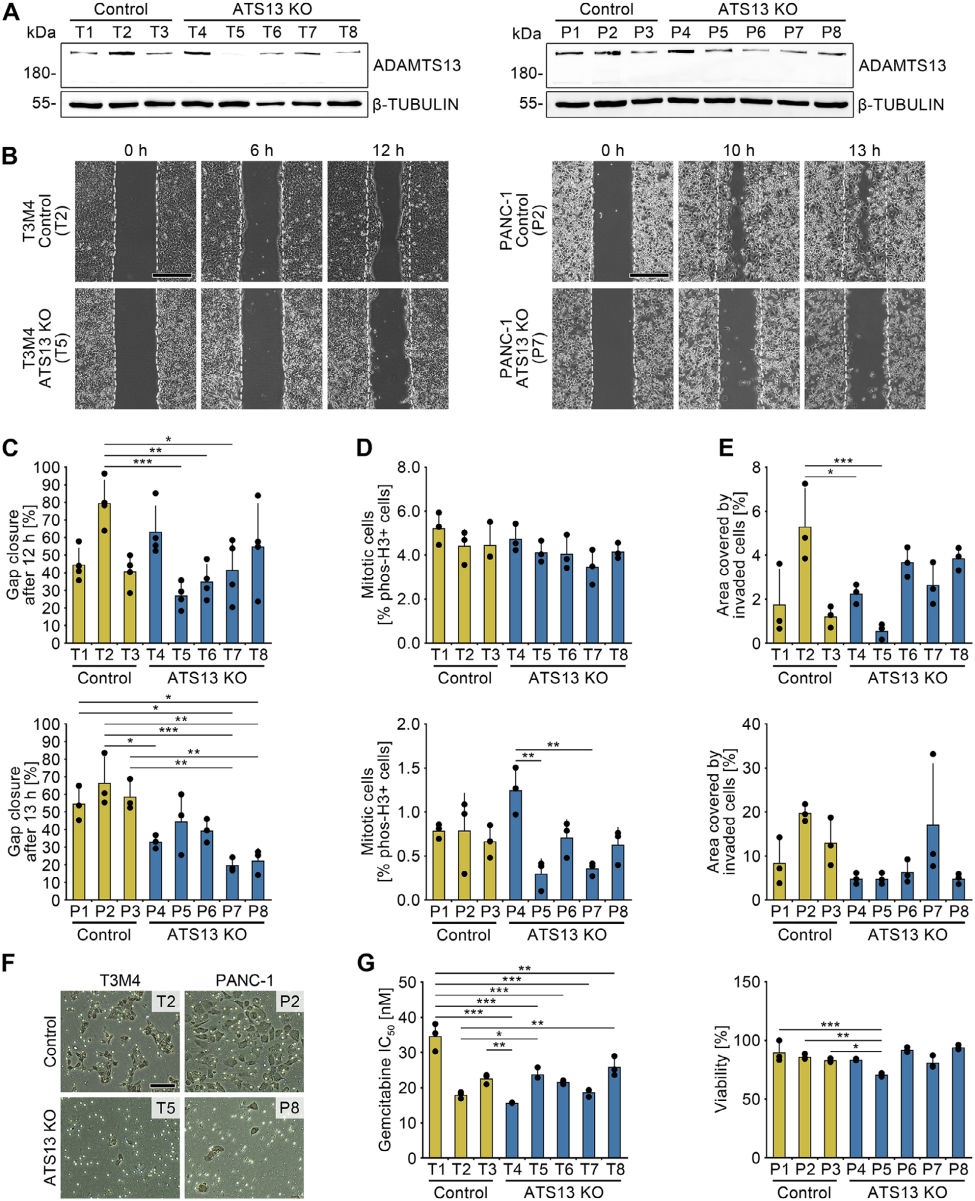
Loss of the metalloprotease ADAMTS13 inhibits migration and invasion in PDAC cells. (A) Immunoblot analysis of ADAMTS13 for validation of ADAMTS13 expression in control and ADAMTS13 knockout (ATS13 KO) T3M4 or PANC‐1 single cell clones. β‐tubulin served as loading control. Molecular weight in kDa. (B) Light microscope image of wound healing migration assay with control and ADAMTS13 knockout T3M4 or PANC‐1 single cell clones after 0, 6, and 12 h or 0, 10, and 13 h, respectively. Dashed lines show the migration front at 0 h. Scale bar 500 µm. *N* = 4 (T3M4) or *n* = 3 (PANC‐1), with 3 technical replicates per experiment. (C) Quantification of percentage gap closure after 12 or 13 h. One‐way ANOVA. (D) Quantification of the percentage of mitotic cells of the single cell clones by phospho‐histone H3 (Ser10) staining. *N* = 3, with 5 (T3M4) or 18 (PANC‐1) evaluated images per experiment. One‐way ANOVA. (E) Matrigel invasion assay with control and ADAMTS13 knockout T3M4 and PANC‐1 single cell clones 72 h after cell seeding. Quantification of the percentage area occupied by cells migrated through the membrane (*n* = 3). One‐way ANOVA. (F) Light microscope image of Matrigel invasion assay. Scale bar 100 µm. (G) MTS assay of control (LentiCRISPRv2) and ADAMTS13 knockout T3M4 and PANC‐1 single cell clones to determine gemcitabine IC_50_ (T3M4) and survival upon treatment with 100 µM gemcitabine (PANC‐1) for 48 h of treatment, respectively. *N* = 3, with three technical replicates per experiment and concentration tested. One‐way ANOVA. *p*‐values ≤ 0.05 (*), *p* ≤ 0.01 (**), and *p* ≤ 0.001 (***) were considered significant. *p‐*values > 0.05 were considered not significant.

CRISPR clones of both cell lines lacking ADAMTS13 expression showed reduced or absent protein levels compared to empty vector controls (Figure 4A). While ADAMTS13 loss had minimal impact on proliferation in T3M4 cells and only a slight effect in PANC‐1 cells (Figure 4D), it markedly impaired migration and invasion in both lines (Figure 4B,C,E,F). Sensitivity to gemcitabine was only modestly and variably affected, particularly at higher concentrations (Figure 4G).

These findings indicate that ADAMTS13 primarily promotes tumor cell motility, with limited influence on proliferation or chemoresistance.

### 2.5 ADAMTS13 Induces a Mesenchymal Phenotype in PDAC Xenograft Tumors

**FIGURE 5:**
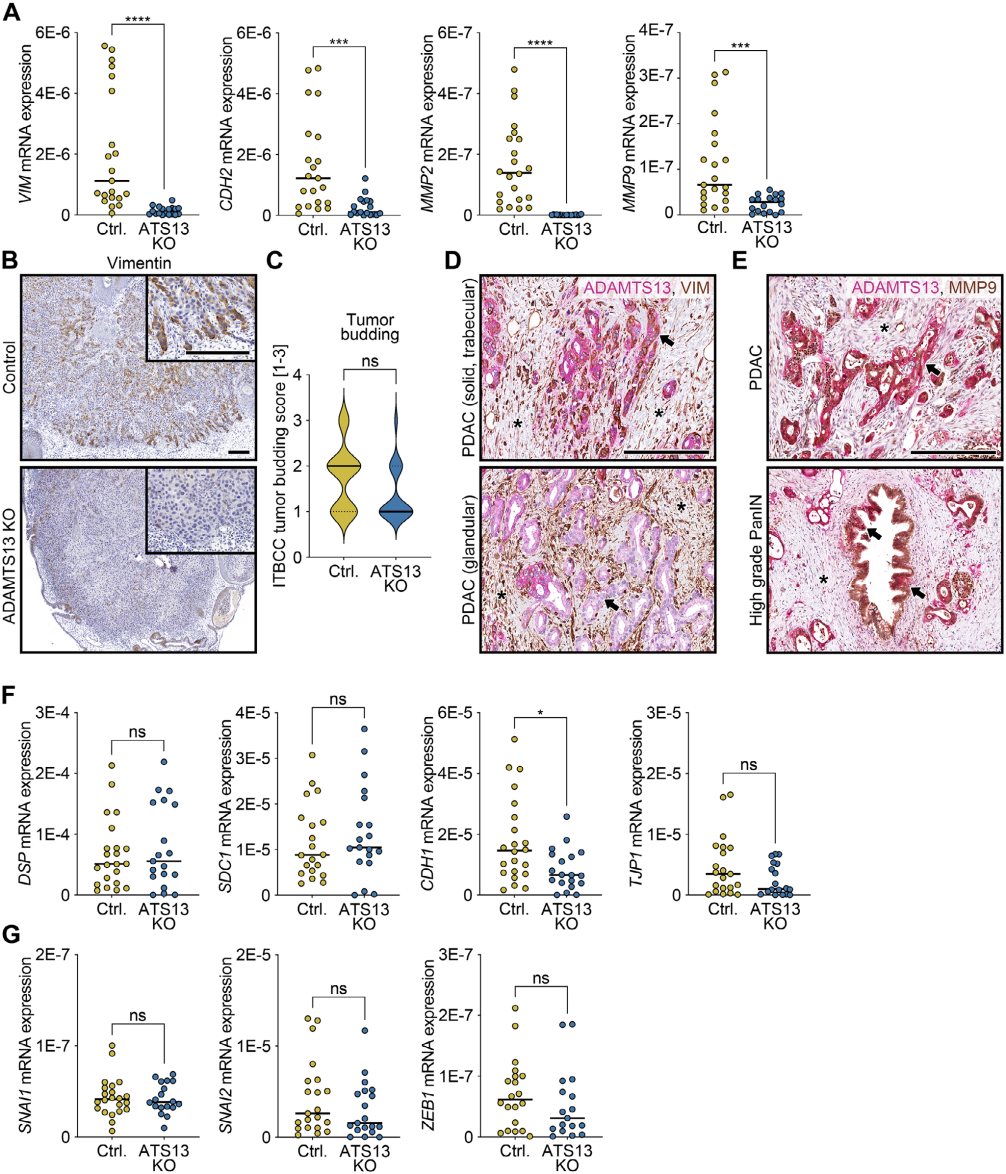
Decreased expression of mesenchymal marker genes upon ADAMTS13 depletion. (A) Semiquantitative real‐time PCR analysis for the mesenchymal markers *VIM*, *CDH2*, *MMP2*, and *MMP9* (A), the epithelial markers *DSP*, *SDC1*, *CDH1*, and *TJP1* (F), the transcription factors involved in EMT *SNAI1*, *SNAI2*, and *ZEB1* (G) of CAM xenograft tumors grown for 11 days and generated from control (LentiCRISPRv2) and ADAMTS13 knockout (ATS13 KO) T3M4 single cell clones (Ctrl., *n* = 22; ATS13 KO, *n* = 19). Each point represents the mean of two technical replicates of a single CAM tumor. Expression was normalized to *RNA18S*. Mann–Whitney or *t*‐test. (B) Immunohistochemical staining for vimentin and (C) quantification of tumor budding based on the international tumor budding consensus conference (ITBCC) of control and ADAMTS13 knockout T3M4 CAM tumors. (D) Immunohistochemical analysis of ADAMTS13 and vimentin (VIM) in representative PDAC tissues. Areas from the same specimen showing tumor cells with a solid, trabecular, or glandular growth pattern are shown (arrows). Asterisks indicate vimentin‐positive stromal cells. (E) Immunohistochemical analysis of ADAMTS13 and MMP9 in representative PDAC tissues. The images illustrate areas from the same specimen displaying either MMP9‐positive high‐grade PanIN or MMP9/ADAMTS13 double‐positive PDAC tumor cells. The arrows indicate cells of the precursor lesions in which a weak positivity for ADAMTS13 is visible. Asterisks indicate stromal cells. (F,G) Semiquantitative real‐time PCR analysis for the epithelial markers *DSP*, *SDC1*, *CDH1*, and *TJP1* (E), the transcription factors involved in EMT *SNAI1*, *SNAI2*, and *ZEB1* (F) of the same CAM xenograft tumors. Each point represents the mean of two technical replicates of a single CAM tumor. Expression was normalized to *RNA18S*. Mann–Whitney or *t*‐test. The median for all measured values within a specific condition is presented as a horizontal line. *p*‐values ≤ 0.05 (*), *p* ≤ 0.001 (***), and *p* ≤ 0.0001 (****) were considered significant. *p*‐values > 0.05 were considered not significant (ns). Scale bars 200 µm.

EMT is a critical step in the metastatic cascade, marked by a dynamic transition from epithelial to mesenchymal marker expression that promotes tumor cell migration and invasion. Given the link between ADAMTS13 and PDAC cell motility, we analyzed EMT‐related gene expression in xenograft tumors derived from wild‐type and ADAMTS13‐deficient cells using the CAM model and semiquantitative real‐time PCR. Despite comparable proliferation rates, ADAMTS13‐deficient cells formed significantly smaller tumors than controls (Figure S13). Expression analysis revealed a significant reduction in the mesenchymal markers vimentin (*VIM*), cadherin 2 (*CDH2*), matrixmetalloproteinase 2 (*MMP2*), and matrixmetalloproteinase 9 (*MMP9*; *p* < 0.0001 each) in ADAMTS13‐deficient tumors compared to wild‐type tumors, with *MMP2* expression almost completely depleted (Figure 5A).

The reduction of vimentin could also be detected by immunohistochemistry within the same xenograft tumors (Figure 5B). In particular, tumor cells located in the tumor periphery showed strong vimentin positivity in controls, whereas ADAMTS13‐deficient tumors showed almost no vimentin staining. The reduced growth of ADAMTS13‐deficient tumors may be due to a decrease in tumor budding, although this result did not reach statistical significance (*p* = 0.0746; Figure 5C). Strikingly, immunohistochemical double staining of ADAMTS13 and vimentin in human PDAC tissues revealed co‐expression of ADAMTS13 and vimentin in tumor cell clusters with an invasive solid, trabecular growth pattern, while vimentin was absent in glandular tumor structures and precursor lesions (Figure 5D). ADAMTS13 and MMP9 were co‐expressed in both precursor lesions and invasive tumor cells, with MMP9 showing a largely uniform expression pattern (Figure 5E). MMP2 expression appeared in a microfocal pattern and did not correlate with tumor growth patterns (Figure S14). While precursor lesions showed low ADAMTS13 and no MMP2, PDAC cells displayed strong ADAMTS13 and occasional MMP2 co‐expression.

The expression of epithelial marker genes desmoplakin (*DSP*) and syndecan‐1 (*SDC1*) exhibited a slight, though not significant, increase, while that of cadherin‐1 (*CDH1*; *p* = 0.0144) and tight junction protein‐1 (*TJP1*) demonstrated a slight reduction (Figure 5F).

Moreover, no significant differences were observed in the expression of the well‐documented EMT transcription factors snail family transcriptional repressors 1 and 2 (*SNAI1* and *SNAI2*) and zinc finger E‐box binding homeobox 1 (*ZEB1*; Figure 5G). In conclusion, xenograft tumors with intact ADAMTS13 expression exhibit a mesenchymal, migratory, and invasive phenotype compared to ADAMTS13‐deficient tumors, consistent with our findings in human PDAC and supporting the association of ADAMTS13 and EMT.

### 2.6 ADAMTS13 Inhibits Vascularization in PDAC Xenograft Tumors

**FIGURE 6:**
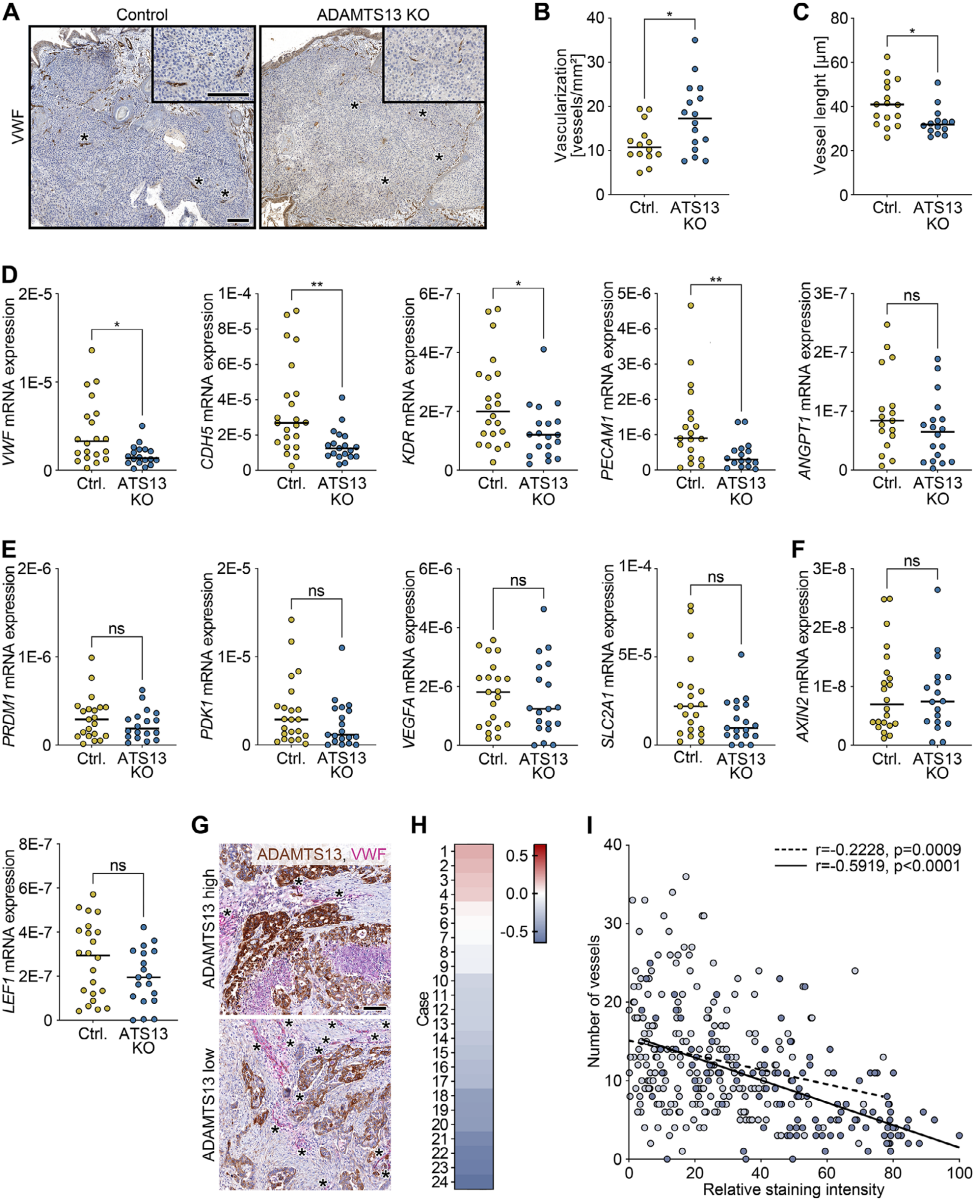
ADAMTS13 inhibits tumor vascularization. (A–C) Immunohistochemical staining for VWF of CAM xenograft tumors (Ctrl., *n* = 22; ATS13 KO, *n* = 19) grown for 11 days and generated from control (LentiCRISPRv2) and ADAMTS13 knockout (ATS13 KO) T3M4 single cell clones. Scale bars 200 µm. Quantification of the number (B) and length (C) of vessels in the tumor. Welch's *t*‐test (vascularization) or Mann–Whitney test (vessel length). (D) Semiquantitative real‐time PCR analysis for the early and late vascular markers *VWF*, *CDH5*, *KDR*, *PECAM1*, and *ANGPT1* in control (LentiCRISPRv2) and ADAMTS13 knockout T3M4 CAM tumors. Each point represents the mean of two technical replicates of a single CAM tumor. Expression was normalized to *RNA18S*. Mann–Whitney test. (E,F) Semiquantitative real‐time PCR analysis for the hypoxia‐responsive genes *PRDM1*, *PDK1*, *VEGFA*, and *SLC2A1* (E) and Wnt target genes *AXIN2* and *LEF1* (F) of the same CAM xenograft tumors. (G) Immunohistochemical analysis of ADAMTS13 and VWF in representative PDAC tissues with low and high ADAMTS13 expression. Scale bar 100 µm. (H) Quantification of ADAMTS13 staining intensity and number of vessels from (G) of 24 patients with digital image analysis and correlation of both parameters. Heat map of the correlation coefficients (ADAMTS13 staining intensity with number of vessels) of the individual cases. (I) Correlogram with Spearman correlation lines of two cases shown in (H) with median (light blue) and high, negative correlation coefficient (blue). Each point corresponds to an evaluated tile of the annotated tumor area. *p*‐values ≤ 0.05 (*) and *p* ≤ 0.01 (**) were considered significant. *p*‐values > 0.05 were considered not significant (ns). The median for all measured values within a specific condition is presented as a horizontal line.

Intratumor vascularization, essential for oxygen and nutrient supply, strongly influences tumor growth, progression, and patient prognosis. Modulating angiogenic factors may offer a therapeutic strategy. To explore the role of ADAMTS13 in in vivo vessel formation, we again utilized the CAM model. In xenograft tumors, ADAMTS13 deficiency resulted in more numerous but smaller vessels (Figure 6A,B,C) and significantly reduced expression of the early and late vascularization markers *VWF*, cadherin‐5 (*CDH5*), kinase insert domain receptor (*KDR*), and platelet and endothelial cell adhesion molecule‐1 (*PECAM1*; Figure 6D).

Given the role of hypoxia in promoting neovascularization via Wnt/β‐catenin signaling [19], we assessed hypoxia‐related genes and Wnt pathway targets. Expression of hypoxia‐related genes PR domain zinc finger protein 1 (*PRDM1*), vascular endothelial growth factor A (*VEGFA*), pyruvate dehydrogenase kinase isoform 1 (*PDK1*), and solute carrier family 2 member 1 (*SLC2A1*) showed slight but non‐significant reductions in ADAMTS13‐deficient tumors (*p* > 0.05 each; Figure 6E), while Wnt target genes *AXIN2* and lymphoid enhancer‐binding factor 1 (*LEF1*) were not significantly altered (Figure 6F).

In line, the in vitro overexpression of ADAMTS13 resulted in reciprocal changes in the expression of most of the genes analyzed (Figure S15). Notably, the induction of hypoxia‐associated genes *PRDM1* and *VEGFA* was also confirmed at the protein level (Figure S16). These changes may be partly mediated by JAK/STAT signaling (Figure S17).

Importantly, in PDAC whole tissue sections (*n* = 24), ADAMTS13 expression was negatively correlated with the vascular density (Figures 6G–I and S18), consistent with xenograft data showing shorter, less mature vessels in ADAMTS13‐deficient tumors.

These data link ADAMTS13 to tumor angiogenesis in both xenografts and human PDAC, suggesting its role in vascular plasticity rather than hypoxia‐driven pathways.

## 3 DISCUSSION

Given its established role in regulating thrombotic events, ADAMTS13 has gained increasing attention in cancer research, particularly in PDAC, where cancer‐associated thrombosis is a common and severe complication [20]. In line with this, reduced ADAMTS13 expression has been linked to advanced disease stages in malignancies such as colorectal cancer [21, 22, 23]. However, findings in brain and prostate tumors remain inconsistent, with no clear correlation to malignancy or metastasis [24]. Beyond its established function in thrombotic regulation, the role of ADAMTS13 in cancer progression, particularly in PDAC, remains poorly understood. In the present study, we report for the first time an aberrant expression of ADAMTS13 in PDAC and its precursor lesions. Notably, ADAMTS13 expression increased progressively from sparse staining in normal ducts and low‐grade precursor lesions to ubiquitous positivity in high‐grade precursor lesions, invasive carcinoma, and metastases. These findings suggest that ADAMTS13 may contribute to carcinogenesis and tumor progression, potentially through autocrine mechanisms.

Since ADAMTS13 is aberrantly expressed in PDAC, its functional role was investigated by assessing proliferation, migration, invasion, and neoangiogenesis following ADAMTS13 knockout. Depletion of ADAMTS13 significantly reduced migration and invasion in vitro. Moreover, ADAMTS13‐deficient xenograft tumors exhibited markedly lower expression of mesenchymal markers *VIM* and *CDH2*, as well as reduced levels of *MMP2* and *MMP9*. While MMPs are not classical mesenchymal markers, their downregulation further suggests a pivotal role for ADAMTS13 in promoting invasive growth behavior. These findings may indicate a link between ADAMTS13 and the acquisition of mesenchymal features, consistent with reports for other ADAMTS family members. For example, ADAMTS1 expression is upregulated in lung carcinoma and associated with EMT both in vitro and in vivo. By analogy with our findings for ADAMTS13, knockdown of ADAMTS1 reduces migration, while overexpression induces N‐cadherin and vimentin expression [25]. ADAMTS12 enhances tumor cell migration in PDAC [26], while ADAMTS15 increases cellular motility without affecting proliferation in breast cancer [27], paralleling the lack of a proliferative effect observed for ADAMTS13 in our study.

Given ADAMTS13's central role in vascular thrombosis [28], a process that promotes neoangiogenesis via ischemia‐ and hypoxia‐driven signaling, we hypothesized that it may influence angiogenic remodeling. Using an avian xenograft model, a well‐established in vivo system for studying angiogenesis, we compared ADAMTS13‐deficient and wild‐type PDAC cells and found that loss of ADAMTS13 led to increased intratumoral vessel formation, but with reduced vascular length, indicative of immature vasculature [29]. This phenotype was linked to downregulation of key angiogenic and angioplastic genes. While hypoxia is a known driver of angiogenesis, our data suggest that the vascular defects observed upon ADAMTS13 loss are only partly hypoxia‐dependent, pointing to a direct role in regulating vascular architecture. Supporting this, analysis of PDAC tissues showed that high ADAMTS13 expression correlated with reduced intratumoral vessel density, mirroring findings from the xenograft model. Together, these results demonstrate for the first time a direct role for ADAMTS13 in modulating tumor angiogenesis in human cancer. We found that high ADAMTS13 expression correlates with improved survival in our human PDAC cohort. The contribution of tumor vascularization in PDAC is controversial: some studies link higher microvascular density to poor prognosis in human and murine models [30, 31], while others associate it with better outcomes, citing increased immune cell infiltration [32] and reduced hypoxia‐driven tumor promotion [33]. Given the marked heterogeneity in PDAC cell and immune cell distribution, prognosis likely reflects the net balance of these pro‐ and anti‐tumor effects, which can vary greatly between patients [30, 34].

The mechanisms driving ADAMTS13 induction in tumor cells remain unclear. Given that PDAC frequently infiltrates adjacent adipose tissue, soluble adipose‐derived factors may contribute to its upregulation. Using a proteome profiler array, we identified the adipokine leptin as an inducer of ADAMTS13 in tumor cells. CFM likewise increased ADAMTS13 expression, an effect partially reduced by a leptin antagonist. Clinically, elevated serum leptin levels are associated with increased PDAC risk [7] and a “fatty pancreas” characterized by intrapancreatic adipocyte deposition correlates with a higher incidence of PDAC [35]. In xenografts, CFM strongly induced ADAMTS13 protein levels, whereas leptin alone caused only a modest increase, indicating that leptin present in CFM contributes to, but does not fully account for, this effect. These findings suggest that additional, yet unidentified CFM components, likely varying between patients, play a major role in ADAMTS13 induction. Identifying these factors will be essential for understanding its regulation in PDAC. Other adipokine‐regulated factors, such as the protease MMP12 and the protease inhibitor SERPINA12, are also expressed in PDAC, suggesting a complex interplay of adipocyte‐derived mediators. These findings indicate that adipocyte‐derived soluble factors may influence both PDAC precursors and established tumors. The heterogeneous ADAMTS13 expression observed within PDAC may reflect differences in local and systemic influences. Locally, adipocytes at the invasive margin can be separated from tumor cells by dense stroma or be in direct contact, leading to distinct interactions [36]. Systemically, adipokines released by distant adipose depots can reach the pancreas via the bloodstream, potentially affecting tumor cells depending on intratumoral vascularization. Moreover, intrapancreatic adipocytes may serve as an additional source of adipokines, contributing to the variable ADAMTS13 distribution at both the invasive front and tumor center.

In conclusion, our data demonstrate for the first time alterative functions of ADAMTS13 in PDAC besides thrombogenesis. We show multifaceted effects of ADAMTS13: on the one hand, there is a tumor‐promoting role by facilitating migration and invasion, induction of mesenchymal markers and matrix metalloproteinases, and tumor growth. These tumor‐associated features may be partly regulated by JAK/STAT signaling. On the other hand, there are prominent anti‐angiogenic effects, without significant alteration of tumor cell proliferation. These data suggest that the anti‐tumor effects of ADAMTS13 outweigh the pro‐tumorigenic features, which may explain the slightly better prognosis in patients with high ADAMTS13 expression in our cohort. A note of caution should be kept in mind. While there is compelling evidence of localized tumor‐promoting effects, such as increased migration and invasion, we also observed alterations in potential downstream factors of ADAMTS13, such as MMP9, that have multifaceted functions in human cancers. MMP9 has been linked to cancer cell migration and invasion. However, opposing characteristics were shown, including reduced angiogenesis and tumor regression [37]. In addition to MMP9, we identified several factors regulated by ADAMTS13 that play a decisive role in the formation of blood vessels and can therefore significantly influence tumor growth. These include VEGF, which is required for almost all aspects of vessel formation. An uninvestigated factor in our study might be the systemic influence of ADAMTS13 in tumor patients, for example, potential negative effects of (micro)thromboembolism in distant organs (lung, heart, brain). While the direct link between ADAMTS13‐mediated vascular alterations or the ADAMTS13‐induced mesenchymal phenotype and improved patient survival requires further analysis, our findings align with previous studies demonstrating that ADAMTS proteases modulate endothelial cell function, tumor angiogenesis, and metastasis [38, 39, 40, 41, 42]. ADAMTS protease activity is typically mediated through substrate cleavage [11, 43]. However, the only known substrate of ADAMTS13, VWF [44] is not expressed in our PDAC cell lines and thus cannot account for the observed effects, suggesting an unknown substrate may be involved. Some ADAMTS family members undergo cellular reuptake via the LRP1 receptor [45, 46, 47, 48], raising the possibility that ADAMTS13 could also act proteolytically inside cells through a similar mechanism.

Altogether, local pro‐ and anti‐tumor effects and systemic effects may be outweighed by another, thus rendering a slightly better prognosis for patients with higher ADAMTS13 expression. These data should be considered when interacting with the blood coagulation system in tumor patients but may also offer novel therapeutic pathways in personalized oncology.

## 4 Materials and Methods

### 4.1 Patient Tissues

All tissue samples were provided by the tissue bank of the University Medical Center Mainz in accordance with the regulations of the tissue biobank and the approval of the ethics committee of the University Medical Center Mainz. For histological analyses, formalin‐fixed and paraffin‐embedded material was used; for RNA analyses, fresh frozen samples were obtained. For CFM preparation, vital visceral adipose tissue was used.

4.2

A TMA was created from formalin‐fixed, paraffin‐embedded tumor cases (*n* = 112, sample size can vary due to cores being lost during sectioning process or due to cores being deemed unevaluable). It comprises two 1 mm cores with tissue of normal ducts, low‐grade PanIN, high‐grade PanIN, tumor center, tumor periphery/invasive margin, and corresponding lymph node metastases. The tumor staging was established according to the eighth edition of the UICC/American Joint Committee on Cancer staging manual [49]. For the exact demographics of the TMA, see Table 1 and [50].

**TABLE 1:** Patient data of the tissue microarray^a^ used for immunohistochemistry (*n* = 112).

		Number of patients (total: *n* = 112)
Gender (female:male)	56:56	
Age (years)	37‐82 (mean: 67, median: 69)	
Tumor size^a^pT	pT1 (< 2 cm)	5
	pT2 (2–4 cm)	73
	pT3 (> 4 cm)	29
	No data	5
Lymph node metastases^a^N	pN0: no regional lymph node metastases	37
	pN1: metastases in 1–3 regional lymph nodes	41
	pN2: metastases in ≥4 regional lymph nodes	33
	No data	1
Distant metastases^a^M	pM0: no distant metastases	15
	pM1: distant metastases	5
	No data	92
Histological grading^a^G	G1: well differentiated	6
	G2: moderately differentiated	62
	G3: poorly differentiated	43
	G4: undifferentiated	1

^a^ The TMA and associated patient data have already been published [50].

### 4.3 Immunohistochemistry and Evaluation

Immunohistochemistry on paraffin‐embedded material was performed on a standardized platform as previously described in detail [50].

The slides were digitized with a NanoZoomer 2.0‐HT scanner (Hamamatsu Photonics, Herrsching, Germany). The evaluation was made with the light microscope type Olympus BX51 (Olympus Corporation, Tokyo, Japan) and software‐based using NDP.view2 (Hamamatsu) and QuPath software [51].

Immunopositivity of ADAMTS13, SERPINA12, and MMP12 was evaluated using the established Allred immunoreactive scoring system: 0–8 (0: negative; 2–3: low; 4–6: medium; 7–8: high) [52]. The Allred score is the sum of positive cells proportion score (0: absent; 1: < 1%; 2: 1%–10%; 3: 11%–33%; 4: 34%–66%; 5: > 67% positive cells) and the intensity of staining score (0: absent; 1: mild; 2: moderate; 3: intense reaction) [50]. A six‐stage scoring system was used to refine the evaluation of the staining intensity to identify minor differences. An intensity score of 0 was defined as a total absence of staining, a score of 1 as weak staining in less than a third of tumor cells, a score of 2 as a weak moderate in less than half of the tumor cells, a score of 3 as diffuse moderate staining without cells with strong staining intensity, a score of 4 as a staining in all tumor cells and strong staining in a subgroup, but not all tumor cells, and a score of 5 as diffuse and strong staining.

Analysis of neovascularization of tumors grown on the CAM was performed as follows: The tumors were outlined using QuPath software to determine the total area. All vessels located within the tumors and at least three cell layers from the tumor margin were measured using NDP.view2. Vessels resting on the CAM and merely extending into the tumor borders were excluded. The vessel number with respect to the tumor area and the mean vessel length were considered measures of tumor vascularization.

To analyze tumor vascularization in relation to ADAMTS13 expression in PDAC sections, immunohistochemical staining for ADAMTS13 and VWF was performed. The tumor was outlined with QuPath, stain vector normalization was performed, and the annotated area was divided into tiles of 800 × 800 pixels. QuPath was used to determine the DAB‐positive (ADAMTS13) pixels for each square with the following threshold values: resolution: very high (0.46 µm/px), channel: DAB, prefilter: Gaussian, smoothing sigma: 0, threshold: 0.6, above threshold: positive, below threshold: unclassified, region: everywhere. Complete vessel cross‐sections were then counted manually for each tile (Figure S12).

For quantification of ADAMTS13‐positive tumor cells in the CAM model, tumors were annotated in QuPath using the simple tissue detection command after stain vector normalization. Cell/nuclei detection was performed using StarDist extension [53]. Tumor cells were classified by nucleus area > 35 µm^2^, and the mean cell DAB staining intensity of tumor cells was calculated per sample.

To evaluate the number of Ki67‐positive cells, the tumors were outlined with QuPath software. Large foci of immune cell infiltrates and large vessels were excluded. The DAB‐positive cells were detected via “analyze > positive cell detection.”

Scoring of tumor budding in CAM tumors was performed as previously described [54].

### 4.4 Protein Isolation

Soluble proteins were extracted using RIPA‐lysis buffer (Santa Cruz) by incubating the cells on ice for 30 min and vortexing every 5 min. The samples were centrifuged at 10,000 g for 10 min at 4°C and the supernatants (soluble proteins) were collected.

For protein extraction from human cryopreserved tissue, 5 µm‐thick sections were cut on a cryostat microtome and placed into homogenization tubes (Tissue homogenizing CKMix, Precellys), mixed with lysis buffer (50 mM Tris‐HCl, 15 mM EGTA, 100 mM NaCl, 1% (v/v) Triton X‐100, 1:100 Halt protease/phosphatase inhibitor cocktail (100 x), Thermo Scientific and homogenized with a Precellys 24 homogenizer for 30 sec at maximum power. Homogenates were centrifuged at full speed for 5 min. The supernatant contained the soluble proteins.

### 4.5 Antibodies

See Table S1.

### 4.6 RNA Isolation and Semiquantitative Real‐Time PCR

Total RNA was extracted with TRI Reagent Solution (Invitrogen) following the protocol provided by the manufacturer. 2 µg of RNA was utilized for cDNA using the High‐Capacity cDNA Reverse Transcription Kit (Applied Biosystems). Semiquantitative real‐time PCR was carried out on a QuantStudio 3 System (Applied Biosystems) using the SYBR Green PCR Master Mix (Applied Biosystems) as reagent. The primers used are listed in Table S2.

### 4.7 Cell Culture

All cell lines were authenticated by short tandem repeat (STR) profiling (Multiplexion, Germany) and routinely tested to confirm they were free of mycoplasma contamination. AsPC‐1, BxPC‐3, PANC‐1 (ATCC, Rockville, USA), and T3M4 (a gift of European Pancreas Center, Heidelberg, Germany) were cultured in RPMI with 10% FBS (heat‐inactivated) and 1% penicillin/streptomycin (10,000 U/mL) at 37°C and 5% CO_2_. HEK293T cells were cultured in DMEM with 4.5 g/L glucose, pyruvate with 10% FBS, and 1% penicillin/streptomycin (10,000 U/mL).

### 4.8 CRISPR/Cas‐Mediated ADAMTS13 Knockout

Two guides targeting exon 3 of *ADAMTS13* were designed using E‐CRISP version 5.4 [55] (see Table S2). The corresponding oligonucleotides were annealed and ligated into LentiCRISPRv2 vector via the *Esp3I* restriction site. Positive clones were validated by Sanger sequencing.

Lentiviruses were obtained by PEI transfection of 3.8 × 10^6^ HEK293T cells seeded the day before in 10 cm cell culture dishes. 6.67 µg LentiCRISPRv2, 5 µg pMDL (pMDLg/pRRE), 5 µg pRSV (pRSV‐Rev), and 3.33 µg pVSV (pMD2.G) were transfected. After 24 h, the culture medium was replaced with 6 mL ultrastructure medium. After another 24 h, the supernatant of transfected HEK293T cells was filtered (0.45 µm). PDAC cells were transduced with 2.5 mL of each viral supernatant (guide 1/2) and 7.5 µL polybrene (10 mg/mL) and incubated for 48 h, selected with puromycin and single cell clones were generated. Single‐cell clones were genotyped using Sanger sequencing.

### 4.9 Migration/Wound Healing Assay

Cell culture inserts (Ibidi, 2‐well) were filled with 90 µL of cell suspension per well, containing 3 × 10⁴ (T3M4) or 2 × 10⁴ (PANC‐1) cells, forming a monolayer overnight. Inserts were removed the next day, and the resulting gap was imaged (Eclipse TS2 microscope, Nikon) at 0, 6, and 12 h (T3M4) or 0, 10, and 13 h (PANC‐1) using a 10x objective and NIS‐Elements 5.20.00 software (Nikon). Gap size was analyzed with ImageJ (v.1.52n, National Institutes of Health, USA) [56] and the wound‐healing‐size tool [57], calculating percentage gap closure over time.

### 4.10 Proliferation Assay

4.4 × 10^5^ cells (T3M4) or 3 × 10^5^ cells (PANC‐1) were seeded on 13 mm cover slips in 12‐well plates. The next day, cells were fixed with 3.7% formaldehyde in PBS for 10 min, washed 3x with PBS, and subsequently blocked with 5% FBS, 0.1% Triton X‐100 in PBS for 30 min at 37°C. The blocking solution was removed, and the primary antibody was added to the cells for 30 min at RT. The anti‐phospho‐histone H3 (Ser10) antibody was diluted in a blocking solution. Afterward, cells were washed 3x with 0.1% Triton X‐100 in PBS and incubated with fluorescence‐labeled secondary antibody and DAPI diluted in 1% FBS, 0.1% Triton X‐100 in PBS for 30 min at RT. After another washing step, the coverslips were mounted with MOWIOL 4–88 mounting medium, dried overnight, and fluorescent micrographs were taken. Using the “analysis particles” option of ImageJ, the number of DAPI and phospho‐histone H3‐positive cells was determined.

### 4.11 Invasion Assay

Matrigel inserts (BioCoat Matrigel Invasion Chambers, 8 µm pore size, Corning) were placed in 12‐well plates and rehydrated with 500 µL RPMI‐medium for 1 h at 37°C. The medium was then replaced with 750 mL of fresh medium. T3M4 or PANC‐1 cells (2.5 × 10⁴) were seeded onto the Matrigel in another 500 µL medium and incubated for 72 h in the incubator.

Matrigel was removed and the membranes were washed 3x with PBS. The cells were fixed with methanol for 20 min at −20°C and stained with Mayer's Hämalaun (AppliChem) for 25 min at RT. After two washes with deionized water, membranes were mounted with Eukitt (Sigma‐Aldrich) and imaged the next day (Eclipse TS2 microscope, Nikon, 10x objective) using the NIS‐Elements 5.20.00 software (Nikon). ImageJ was used to quantify the membrane area and the migrated cell area.

### 4.12 Cytotoxicity/MTS Assay

In a 96‐well plate, 7.5 × 10^3^ (T3M4 or PANC‐1) cells per well were seeded in 50 µL RPMI‐medium. The next day, cells were treated with gemcitabine (0.1 nM–1 µM) for 48 h. After incubation, the medium was replaced with 120 µL MTS solution (CellTiter 96 AQueous One Solution Cell Proliferation Assay [MTS], Promega) diluted 1:5.8 in RPMI‐medium and incubated for 2 h at 37°C. 80 µL per well was transferred to a new 96‐well plate. Absorption was measured at 492 nm using a Spark plate reader (Tecan).

### 4.13 Production of Conditioned Fat Medium

For the preparation of CFM, human visceral adipose tissue was cultured in RPMI‐medium. 1 g of adipose tissue was cultured in inserts (Falcon PET membranes 0.4 µm, Corning) on top of 1.5 mL medium in 6‐well plates for 24 h. The CFM was stored at −20°C.

### 4.14 Adipokine Treatment

AsPC‐1, BxPC‐3, PANC‐1, or T3M4 cells were seeded in culture dishes. The next day, the culture medium was replaced by RPMI‐medium without FBS for 16 h. In initial experiments, the starvation medium was replaced with RPMI‐medium supplemented with 10 ng/mL leptin or CFM (diluted 1:6 to obtain a leptin concentration of 10 ng/mL) and incubated for 10 or 24 h. In a second series of experiments, cells were treated with CFM alone or in combination with 100 or 500 ng/mL leptin antagonist or control medium (Ctrl.) for 24 h.

### 4.15 Proteome Profiler Assay

Both proteome profiler arrays, the human protease/protease inhibitor array and the human XL oncology array (R&D Systems), were carried out according to the manufacturer's instructions for protein lysates. For the human protease/protease inhibitor array, BxPC‐3 and T3M4 cells were seeded and treated with 10 ng/mL leptin (see 4.14) or RPMI‐medium as control for 24 h. For the human XL oncology array, PANC‐1 and T3M4 cells were seeded and transfected with the control vector (pcDNA3) or ADAMTS13 overexpression vector (pcDNA3_ADAMTS13) and incubated for 48 h.

### 4.16 CAM Assay

The CAM model is a well‐established in vivo system that, due to its natural immunodeficiency until embryonic day 10 and dense vascularization, provides an ideal environment for studying both angiogenesis and tumor cell invasion. Fertilized chicken eggs (VALO Biomedia, Osterholz‐Scharmbeck Germany) were processed as previously described [58]. On Day 7, 6 × 10⁶ cells were resuspended in 25 µL RPMI and 25 µL Matrigel (Corning), then seeded onto the CAM. On Day 17, cells were treated with RPMI‐medium, 10 ng/mL leptin, or CFM (diluted 1:6.7 to obtain a leptin concentration of about 10 ng/mL). The tumor was collected, size and weight were determined, followed by fixation in formalin for further histological analysis. A small piece of the tumor was previously frozen in liquid nitrogen for RNA isolation.

### 4.17 Statistical Analysis

Statistical analysis of the data was performed using GraphPad Prism (v.9.4.0 (673) GraphPad Software, San Diego, California, USA, www.graphpad.com) and MedCalc (v.22.021 MedCalc Software, Ostend, Belgium, www.medcalc.org). Statistical tests for each experiment are indicated in the figure legends. The analysis was performed on the averages of biological replicates.

## Author Contributions

M.M.G and H.R.W conceptualized the study. S.A., H.R.W, and C.E. visualized the study. M.M.G, H.R.W, and A.H. supervised the study. S.A., N.M., N.H., H.R.W., C.E., S.M., and A.H. performed the investigation. S.A., H.R.W, P.M., and C.E. did the formal analysis. M.M.G, H.R.W, S.A., and H.D wrote the manuscript. M.M.G., W.R., and S.H. handled resources. All authors have read and approved the final manuscript.

## Funding

This study was supported by the German Research Foundation (Project Numbers 318346496, SFB1292/2 TPQ1, TP22, and GA1818 2–3).

## Conflicts of Interest

The authors declare no conflicts of interest.

## Ethics Statement

The study was approved by the ethics committee of the University Medical Center Mainz (approval no. 2019–14390; State of Rhineland‐Palatinate Medical Chamber). A written informed consent was obtained from all patients.

## Supporting information




**Supporting File 1**:mco270462 sup 0001 SuppMat.docx

## Data Availability

The data from this study are available upon reasonable request from the corresponding author.
