# Supplementary material for: Aberrant Expression of A Disintegrin and Metalloproteinase With Thrombospondin Motifs 13 (ADAMTS13) in Pancreatic Cancer Leads to Dichotomic Functions
Source: MedComm (2020). 2025 Nov 6;6(11):e70462. doi: 10.1002/mco2.70462 (PMC12592688; doi:10.1002/mco2.70462)
Supplement: Supplementary file 1 — Supporting File 1:mco270462 sup 0001 SuppMat.docx [file MCO2-6-e70462-s001.docx]

**Aberrant Expression of A Disintegrin and Metalloproteinase with Thrombospondin Motifs 13 (ADAMTS13) in Pancreatic Cancer Leads to Dichotomic Functions**

**Stephanie Allmang^1,#^, Hagen R. Witzel^1,#^, Anne Hausen^1,*^, Simone Marquard^1^, Christoph Eckert^1,2^, Nicole Marnet^1^, Nina Hörner^1^, Philipp Mayer^3^, Stefan Heinrich^4,5^, Hien T. Dang^6^, Wilfried Roth^1^, and Matthias M. Gaida^1,2,7,^**^*^

^1^Institute of Pathology, University Medical Center Mainz, JGU-Mainz, 55131 Mainz, Germany

^2^TRON, Translational Oncology at the University Medical Center, JGU-Mainz, 55131 Mainz, Germany

^3^Clinic for Diagnostic and Interventional Radiology, University Hospital Heidelberg, 69120 Heidelberg, Germany

^4^Department of Surgery, University Medical Center Mainz, JGU-Mainz, 55131 Mainz, Germany

^5^Department of Surgery, Mutterhaus Klinik, Medical Campus Trier, 54290 Trier, Germany

^6^Department of Surgery, Department of Surgical Research, Thomas Jefferson University, Philadelphia, Pennsylvania, USA.

^7^Research Center for Immunotherapy, University Medical Center Mainz, JGU-Mainz, 55131 Mainz, Germany

^#^These authors have contributed equally.

***Correspondence:** [Anne.Hausen@unimedizin-mainz.de](mailto:Anne.Hausen@unimedizin-mainz.de) (AH), [Matthias.Gaida@unimedizin-mainz.de](mailto:Matthias.Gaida@unimedizin-mainz.de) (MMG)

**Supplemental information**


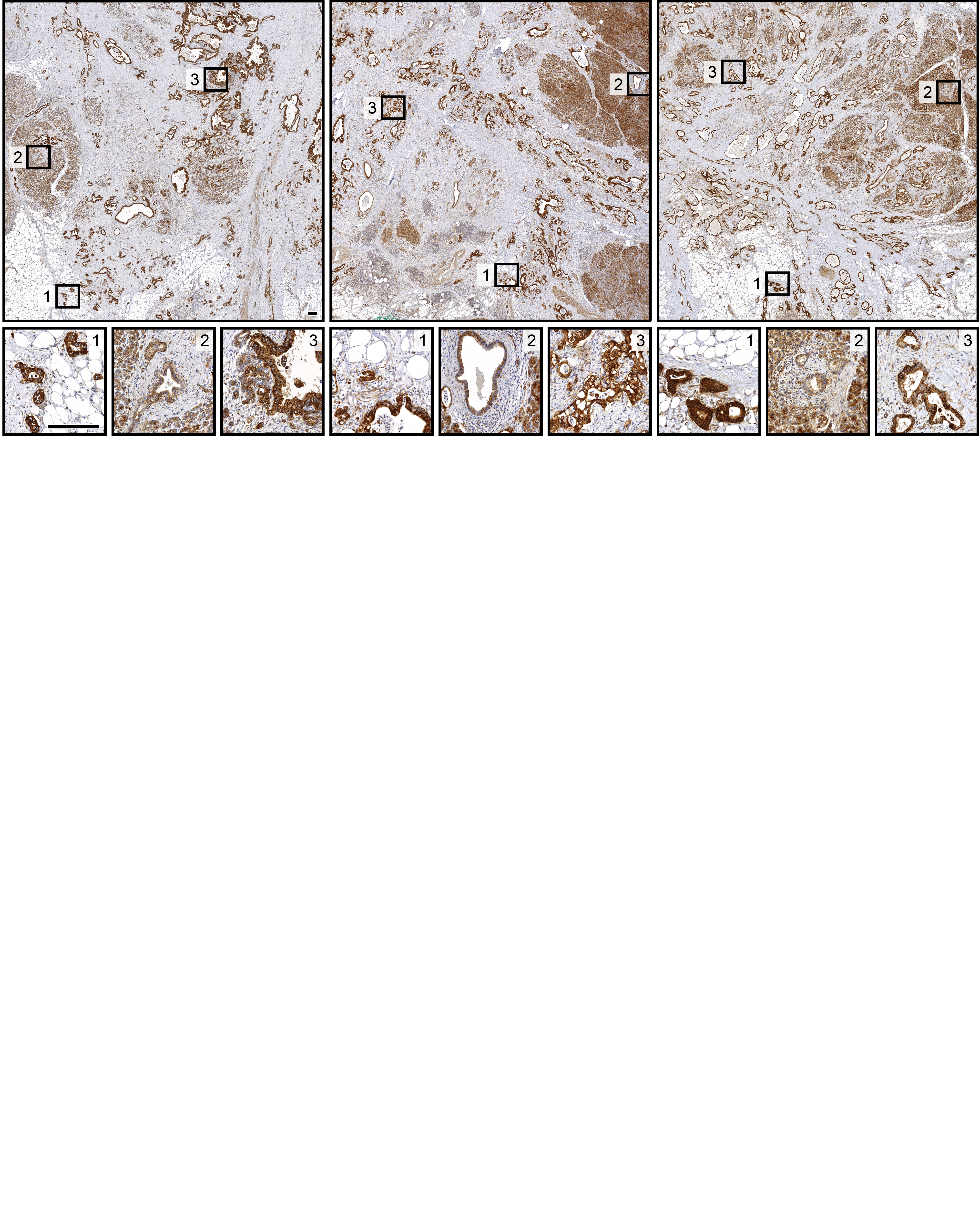


**FIGURE S1** Expression and localization of ADAMTS13 in PDAC. Immunohistochemical analysis on PDAC whole tissue slices from three different patients to investigate the expression of ADAMTS13 at the invasion margin and in the tumor center. The magnified areas show ADAMTS13 expression in tumor cells adjacent to peripancreatic adipocytes (1), normal duct epithelial cells (2), and tumor cells in the center of the tumor. Scale bars 200 µm.


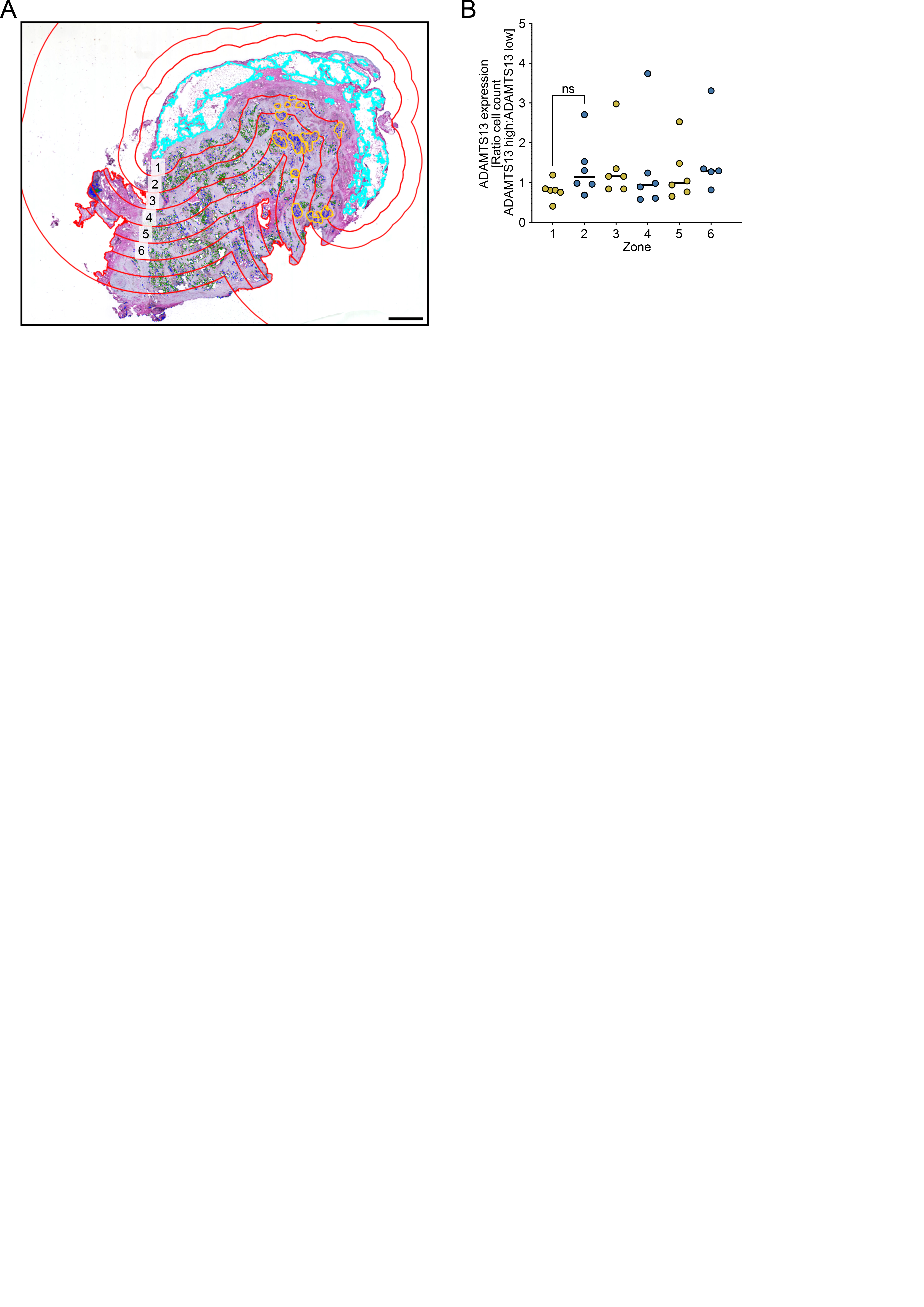


**FIGURE S2** Quantification of ADAMTS13 expression as a function of tumor cell localization. PDAC whole tissue sections were immunohistochemically stained with ADAMTS13 and subsequently digitized. The initial step involved the annotation of the entire tissue sample, in addition to the adipose tissue, for subsequent analysis. The annotation of the adipose tissue (blue outline) served as a reference point for determining the invasion margin. From the adipose tissue, concentric rings were delineated at progressively increasing distances from the adipose tissue. The tumor cells within the aforementioned rings were then identified via ADAMTS13 staining. Cells exhibiting an ADAMTS13 expression level of ≤0.3 AU (arbitrary unit) were considered negative. The median staining intensity of each stained slide was established as the threshold for discriminating between ADAMTS13-high and ADAMTS13-low tumor cells. Scale bar 300 µm. **(A)** Illustrative example of the methodological procedure utilizing a stained whole tissue section. Areas comprising clusters of non-neoplastic acinus cells were excluded from the analytical process and are indicated by an orange outline. The distinct zones in which ADAMTS13 expression was determined are numbered in consecutive order. **(B)** Quantification of ADAMTS13 expression in the various zones (1-6). Six sections were analyzed. The number of ADAMTS13-high and ADAMTS13-low tumor cells was determined for each zone, and the ratio was calculated (high:low). Each data point represents a single ratio of the indicated zone of one sample. The evaluation was conducted using QuPath.^1^ Kruskal-Wallis test did not identify any statistically significant differences in ADAMTS13 expression between the various zones. P-values >0.05 were considered not significant (ns).

**
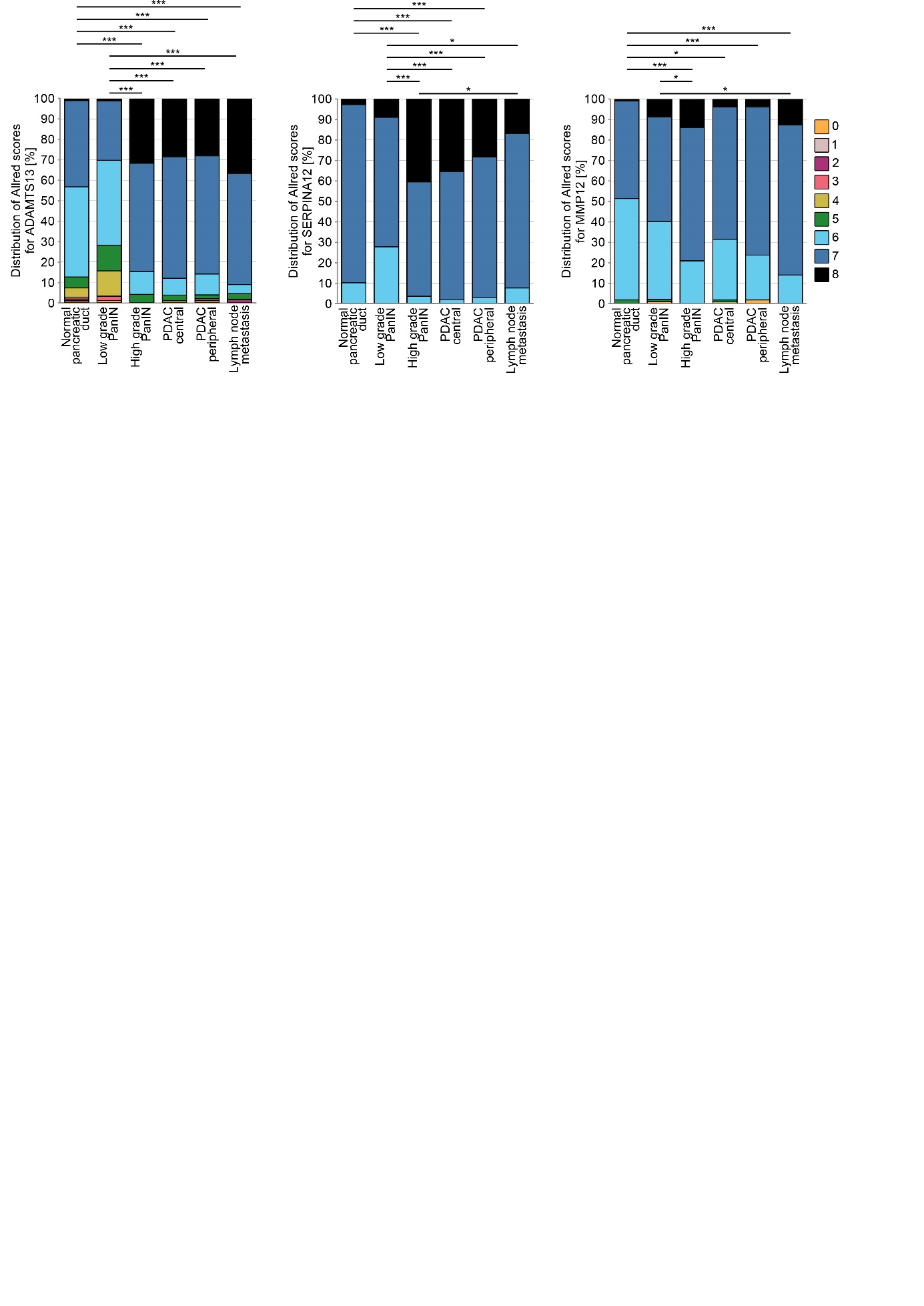
FIGURE S3** Induction of ADAMTS13 expression during the development and progression of PDACs. Quantification of the PDAC-TMA staining for ADAMTS13, SERPINA12, and MMP12. Results are presented as Allred scores (intensity score + proportion score 0, 2-8). Kruskal-Wallis test. P-values ≤0.05 (*), p≤0.001 (***) were considered significant. P-values >0.05 were considered not significant.

**
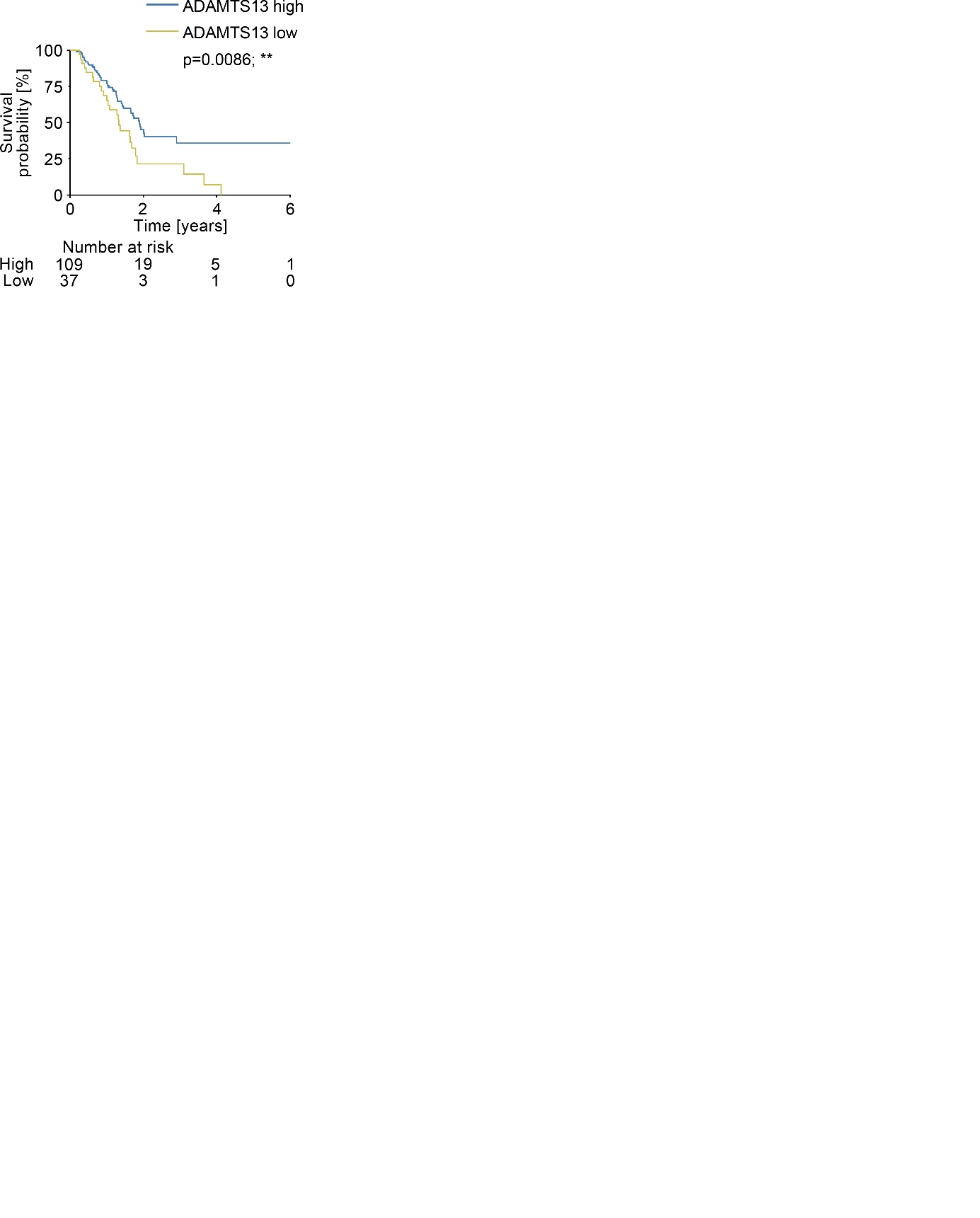
**

**FIGURE S4** Expression analysis of ADAMTS13 of the TCGA-PAAD cohort. Only patients with PDAC as histologic subtype and without proven M1-status were selected for further analysis according to Raphael et al.^2^ Normalized expression values of ADAMTS13 were stratified in two groups based on its lower quartile in low and high ADAMTS13. Kaplan-Meier survival curve, numbers at risk and analysis by log-rank test are shown. P-value ≤0.01 (**) was considered significant.

**
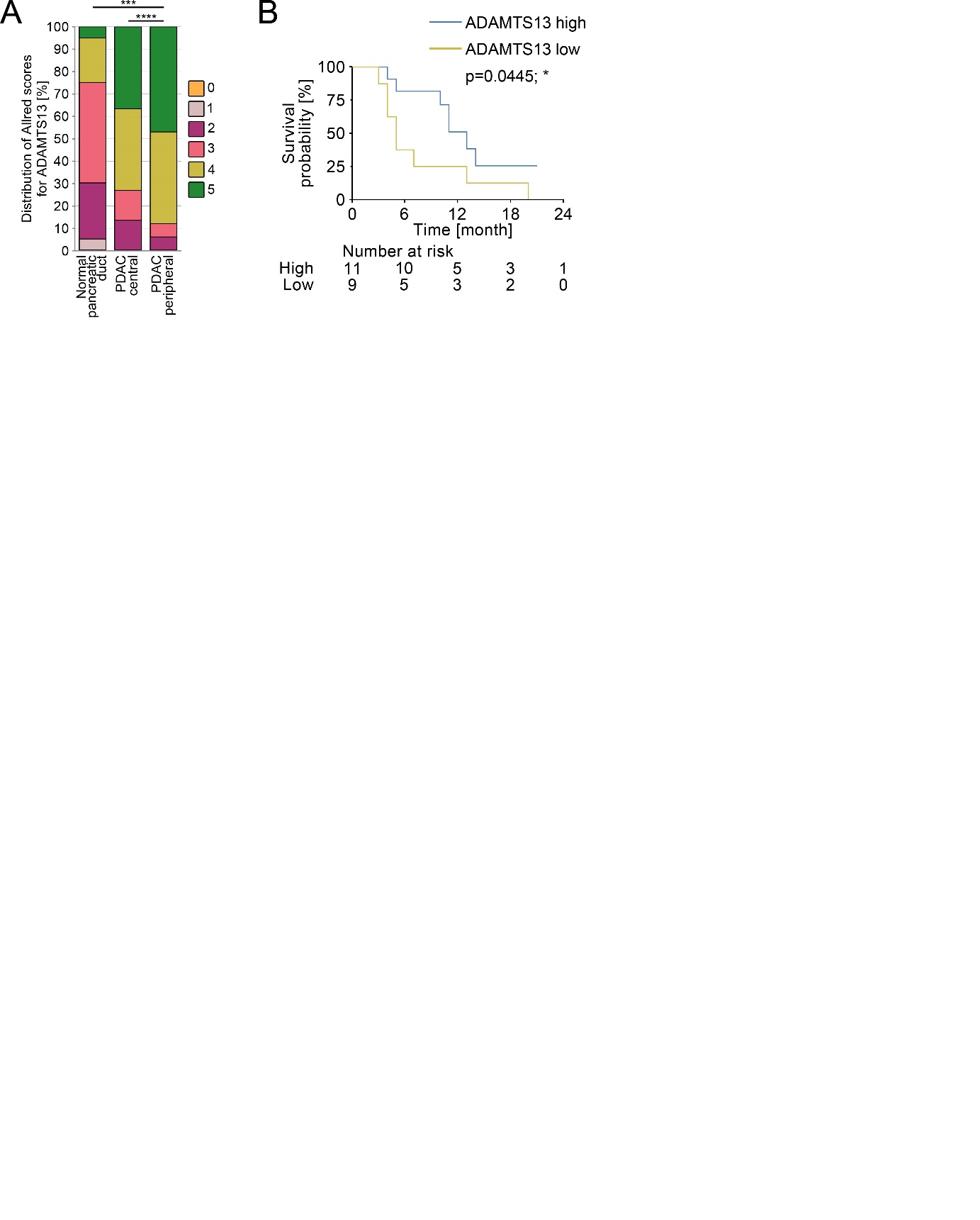
**

**FIGURE S5** The expression levels of ADAMTS13 were analyzed in a PDAC TMA, which comprised tissue from the center and the periphery of the tumor, as well as non-neoplastic adjacent tissue from 41 patients who had undergone diverse neoadjuvant chemotherapy regimens. However, it should be noted that only 20 cases had an evaluable peripheral tumor core due to preparational reasons. (**A)** The ADAMTS13 staining intensity was evaluated in cores of non-neoplastic adjacent tissue, cores from the central and periphery of the tumor using a sensitivity scoring system (0-5). Kruskal-Wallis test. **(B)** Comparison of Kaplan-Meier survival curves between patients exhibiting high and low ADAMTS13 expression in peripheral tumor cores (≥4; n=11 vs. <4; n=9). Gehan-Breslow-Wilcoxon test. P-value ≤0.05 (*), p≤0.001 (***), p≤0.0001 (****) were considered significant.


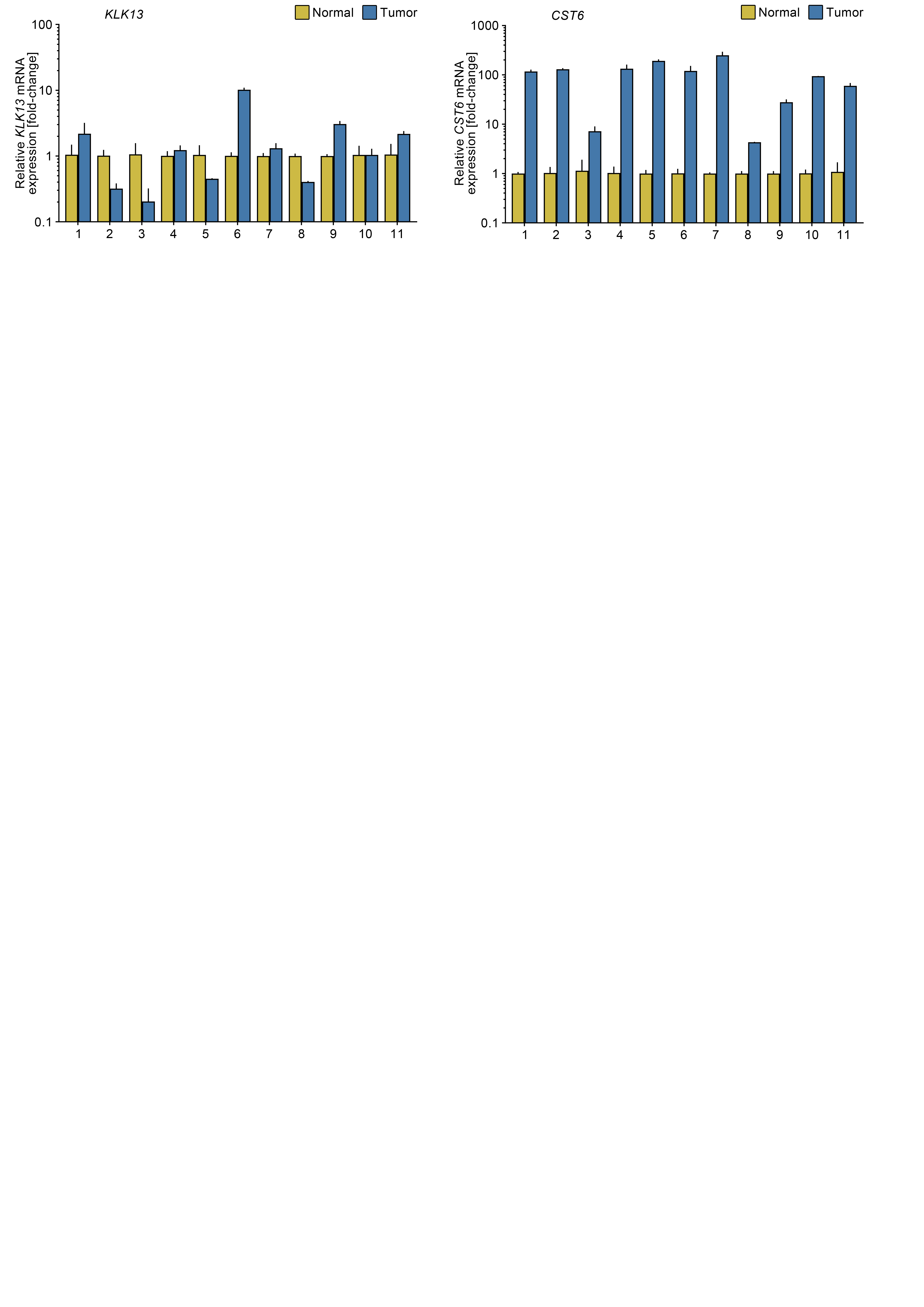


**FIGURE S6** Expression of *KLK13* and *CST6* in PDAC tissue. Semiquantitative real-time PCR analysis for the protease inhibitors *CST6* and *KLK13* in a collective of 11 PDAC (tumor, blue) and the respective adjacent non-neoplastic tissues (normal, yellow). Each bar represents the mean and the respective standard deviation of two technical replicates. Expression was normalized to *RNA18S*. Relative expression in the respective normal tissues was set to 1.


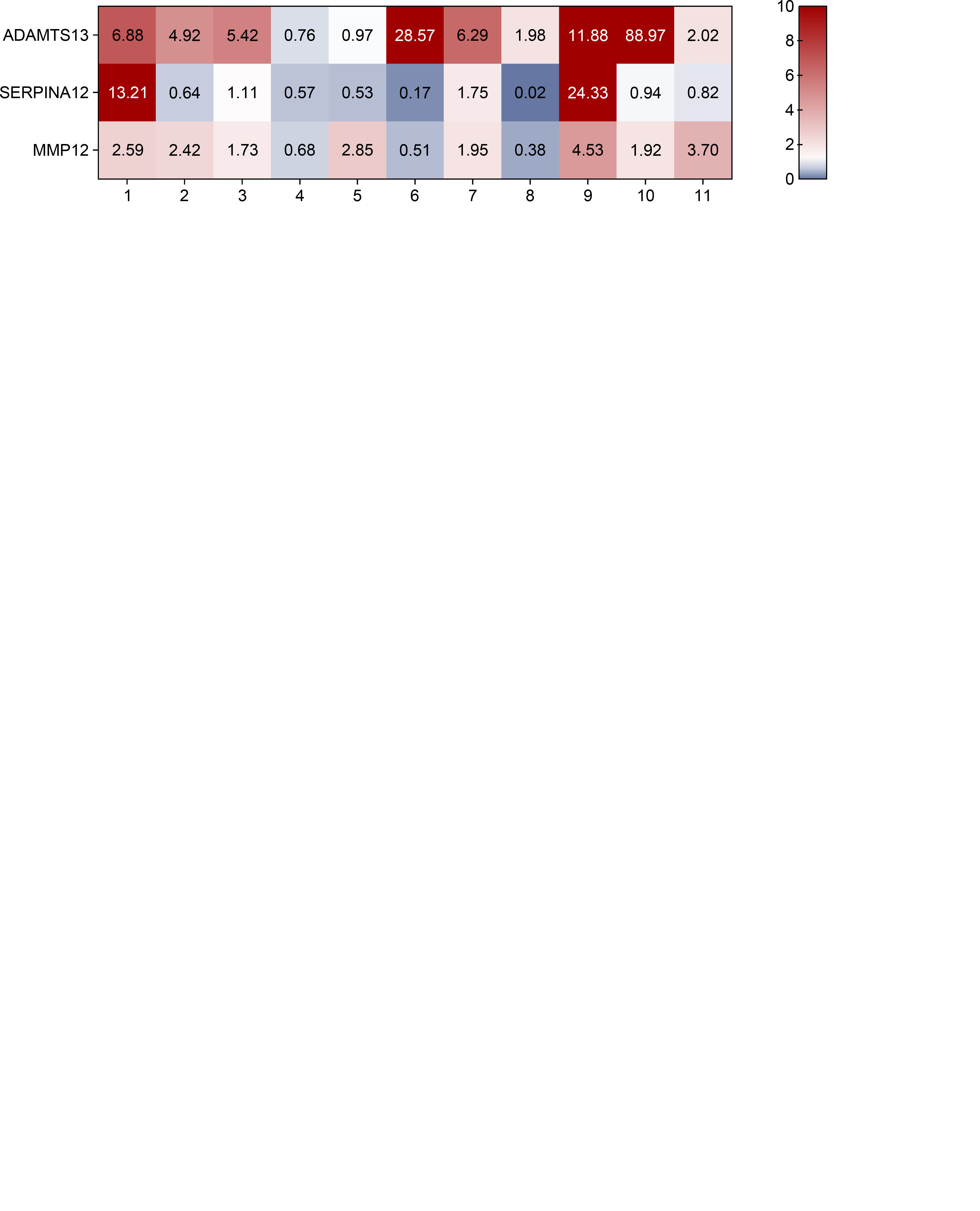


**FIGURE S7** Quantification of the immunoblots depicted in Figure 2B. The intensity of the various ADAMTS13, SERPINA12, and MMP12 bands was determined with the ImageJ software (v.1.52n, National Institutes of Health, USA)^3^ and subsequently divided by the value determined for the loading control VINCULIN. The values provided represent the ratio of the protein of interest to VINCULIN.

**
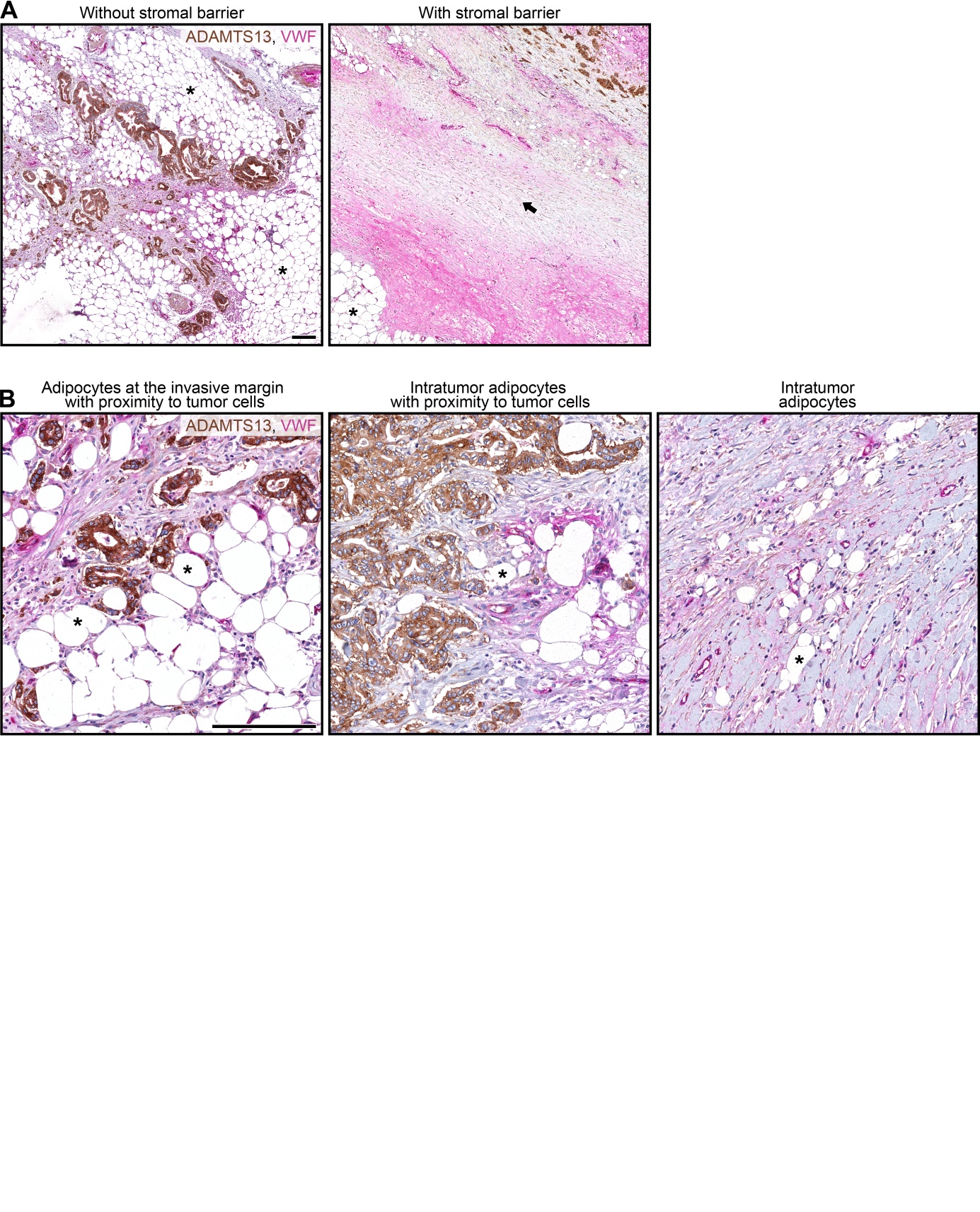
**

**FIGURE S8.** Frequency of a stromal barrier and intrapancreatic adipocytes in PDAC. Immunohistochemical analysis of ADAMTS13 and VWF in representative PDAC whole tissues sections. **(A)** A total of 28 PDAC whole tissue sections were examined to determine whether a direct interface was present between tumor cells and adipocytes of the peripancreatic adipose tissue at the invasive margin, or whether an intervening stromal barrier separated the two. The majority of cases exhibited a robust stromal barrier (64 % (18/28)). **(B)** In addition, the presence of intratumor adipocytes was also examined. A distinction was made between two categories of adipocytes: those in direct contact with tumor cells (50 % (14/28)) and those embedded in stroma (68 % (19/28)). It is noteworthy that intratumor adipocytes, despite their prevalence, are frequently situated within the stroma and lack direct contact with tumor cells. Moreover, intratumor adipocytes are typically scarce and, when present, occur in small clusters with a limited number of cells. Adipocytes are indicated with asterisks, the stromal barrier with an arrow. Scale bar 200 µm.


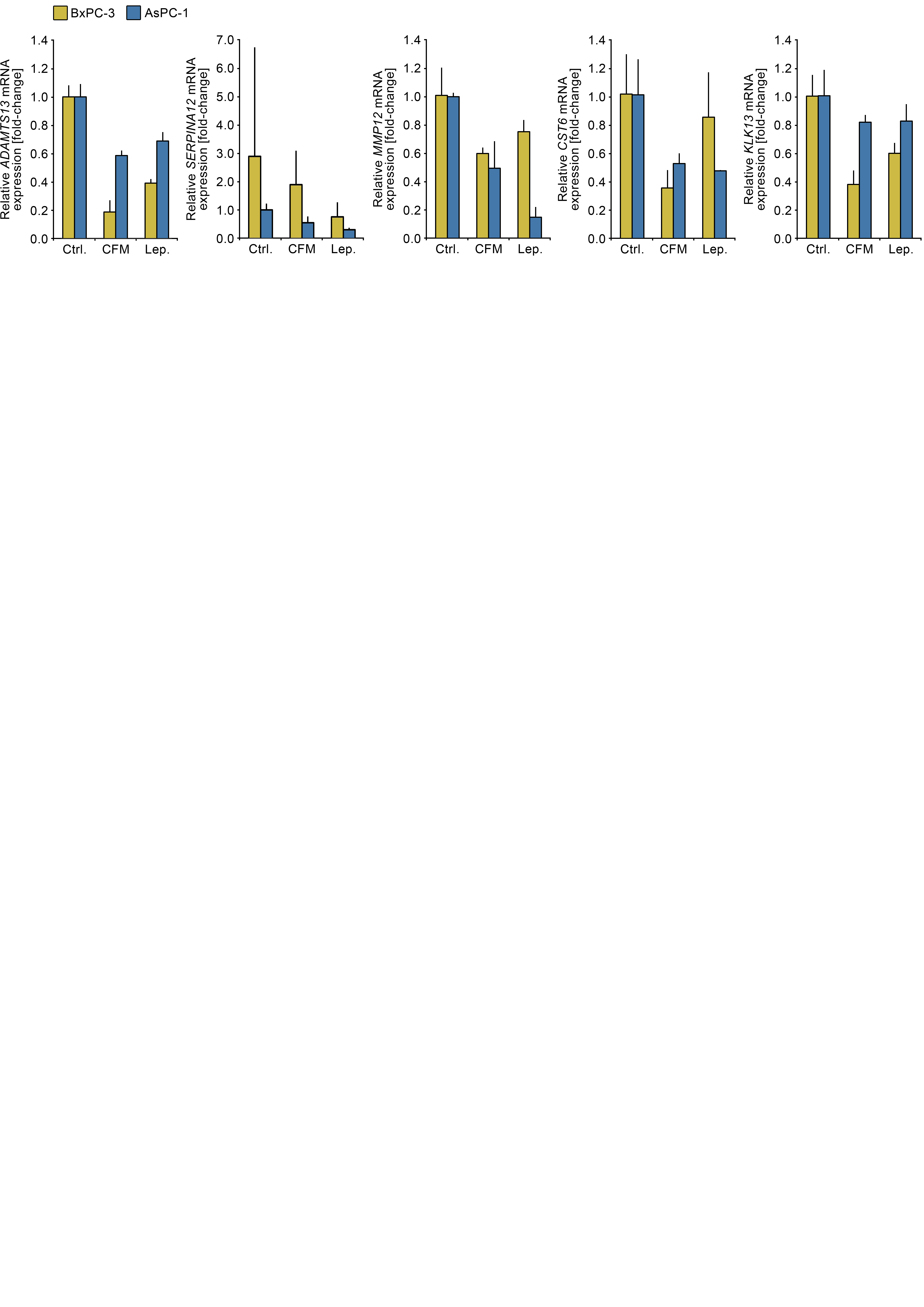


**FIGURE S9** Validation of leptin-regulated factors in PDAC. Semiquantitative real-time PCR analysis for selected proteases and protease inhibitors in BxPC-3 or AsPC-1 cells either treated with 10 ng/ml leptin (Lep.), conditioned fat medium (CFM) or vehicle (Ctrl.) for 24 h. Each bar represents the mean and the respective standard deviation of two technical replicates. Expression was normalized to *RNA18S*. Relative expression in the vehicle treated groups was set to 1.


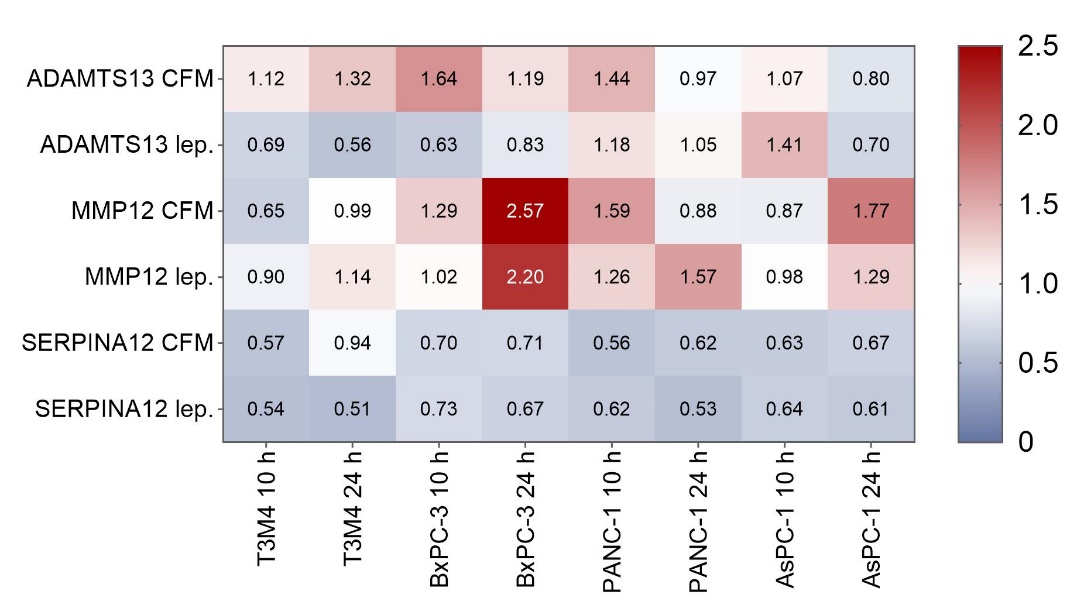


**FIGURE S10** Quantification of the immunoblots depicted in Figure 3E. The intensity of the various ADAMTS13, MMP12, and SERPINA12 bands was determined with the ImageJ software (v.1.52n, National Institutes of Health, USA)^3^ and subsequently divided by the value determined for the loading control VINCULIN. The values provided represent the ratio of the respective treatment condition (CFM or lep.) to the control treatment for each cell line.


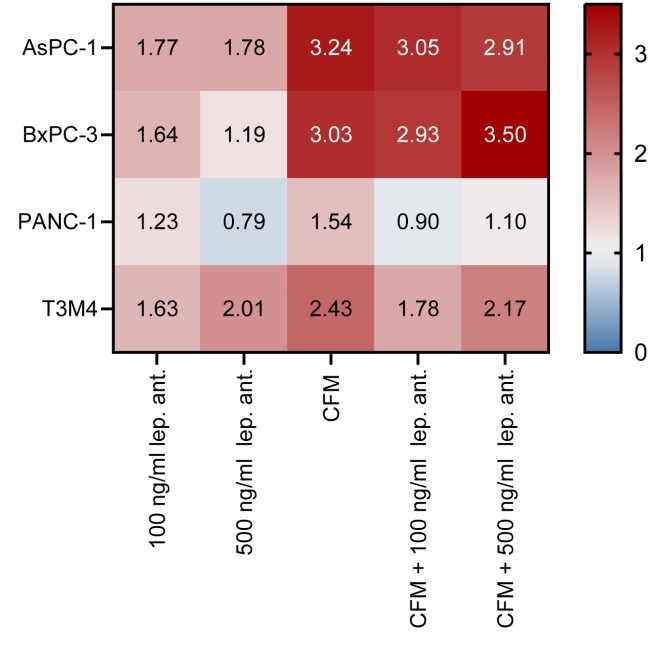


**FIGURE S11** Quantification of the immunoblots depicted in Figure 3F. The intensity of the various ADAMTS13 was determined with the ImageJ software (v.1.52n, National Institutes of Health, USA)^3^ and subsequently divided by the value determined for the loading control VINCULIN. The values provided represent the ratio of the respective treatment condition (leptin antagonist -,+,++ and/or CFM) to the control treatment (leptin antagonist -, CFM -) for each cell line.


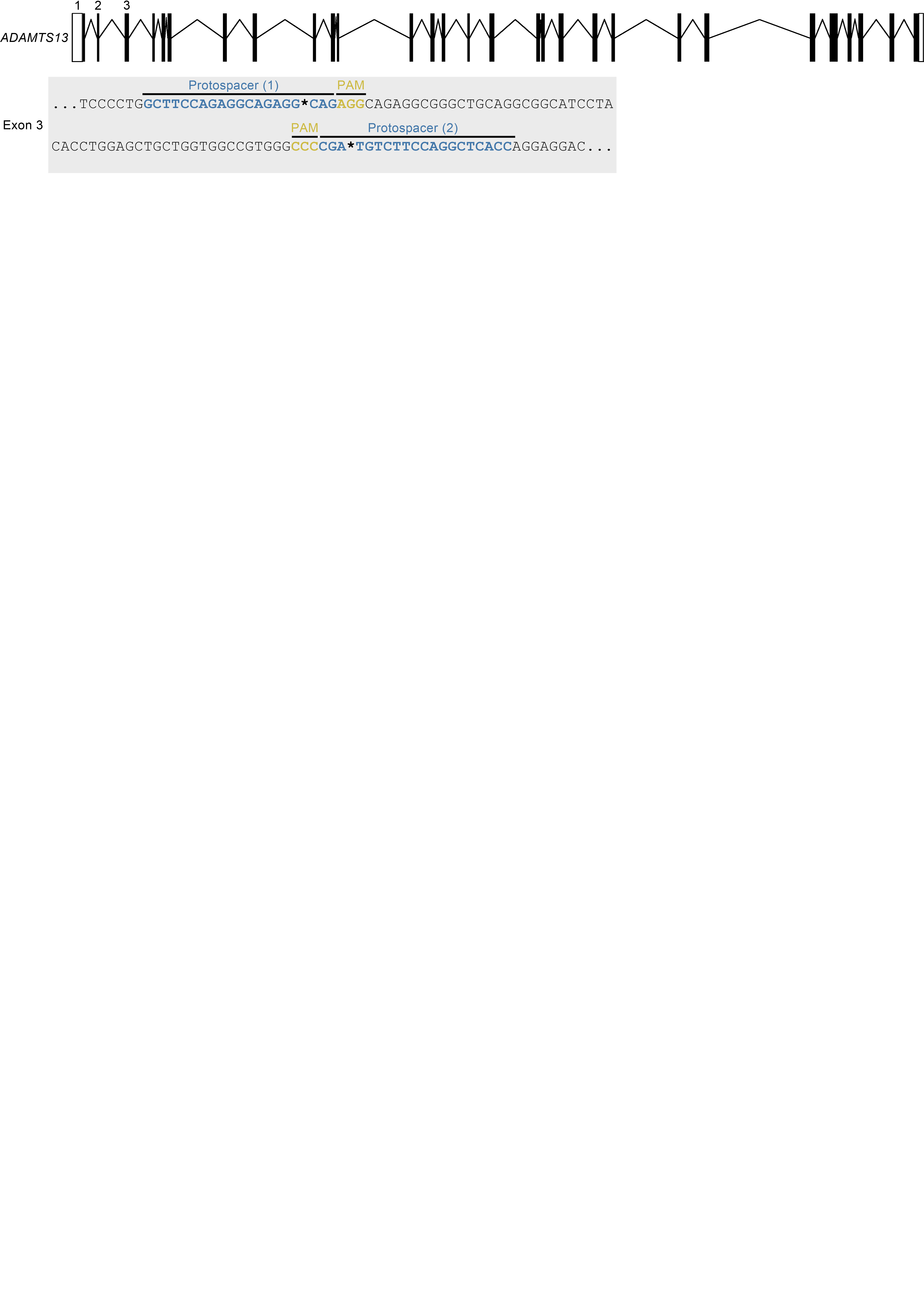


**FIGURE S12** ADAMTS13 knockout strategy. Schematic representation of the CRISPR/Cas-mediated ADAMTS13 knockout strategy in cell culture. Exon-intron structure of the *ADAMTS13* gene and partial sequence of exon 3 with protospacers (blue) and protospacer adjacent motifs PAM (yellow) of the two guide RNAs. The asterisks mark the locations of the induced double-strand breaks.


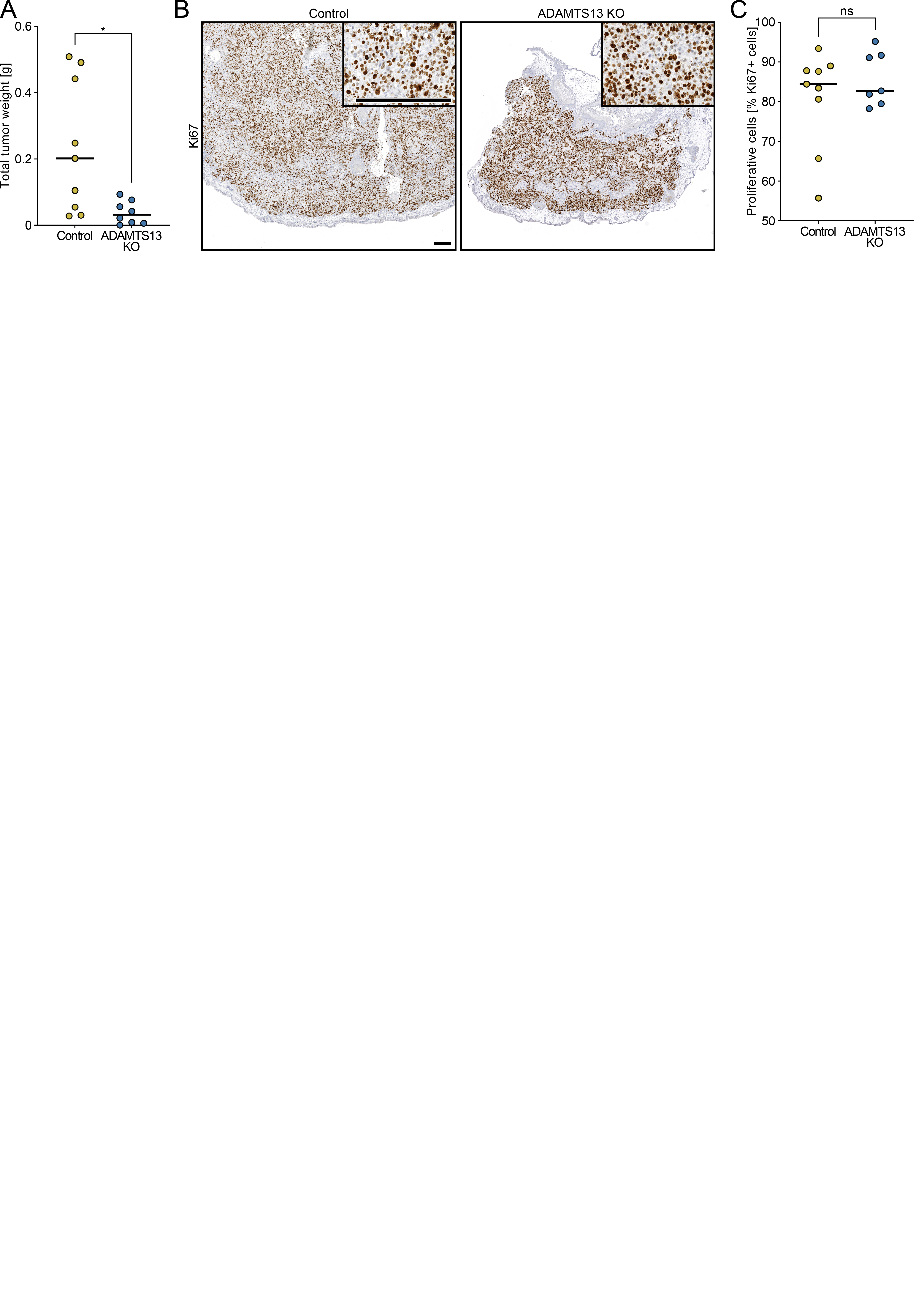


**FIGURE S13** Reduced growth of ADAMTS13 depleted xenograft tumors. **(A)** Total weight of isolated xenograft tumors grown for 11 days and generated from control and ADAMTS13 knockout T3M4. Welch’s t-test. **(B and C)** Immunohistochemical staining for Ki67 of the same tumors and (C) quantification of Ki67 positive cells. Mann-Whitney test. P-values ≤0.05 (*) were considered significant. P-values >0.05 were considered not significant. Scale bars 200 µm.


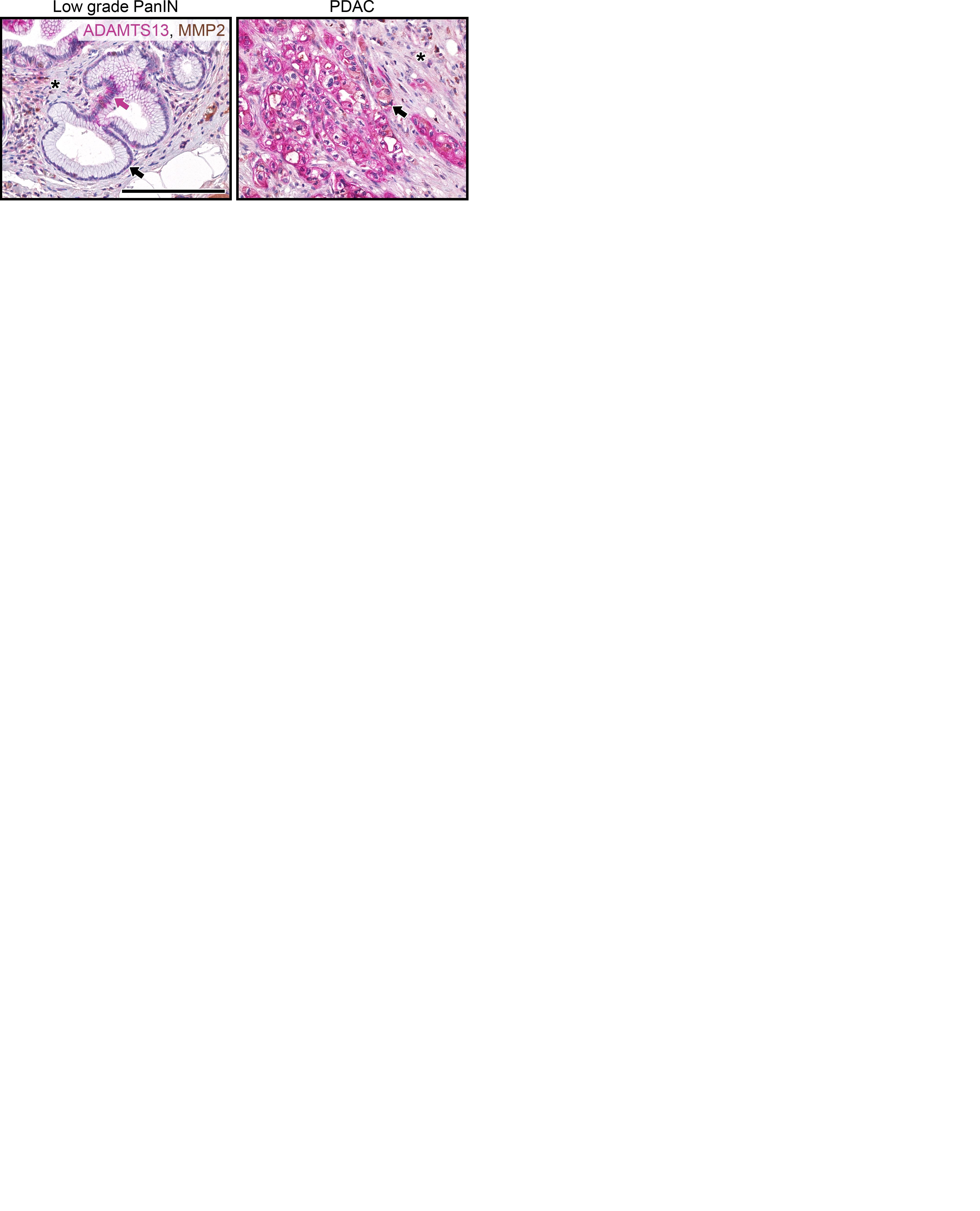


**FIGURE S14** MMP2 expression is observed in a singular manner in PDAC. Immunohistochemical analysis of ADAMTS13 and MMP2 in representative PDAC tissues. Precursor lesions (left: black, purple arrow) exhibit low levels of ADAMTS13 expression, which is subsequently observed in a limited number of cells (purple arrow). These cells are negative for MMP2. In contrast, PDAC cells exhibit robust ADAMTS13 expression and sporadic positivity for MMP2 (right: black arrow). However, this MMP2 expression does not correlate with the different growth patterns of the tumor. Asterisks indicate stromal cells. Scale bars 200 µm.


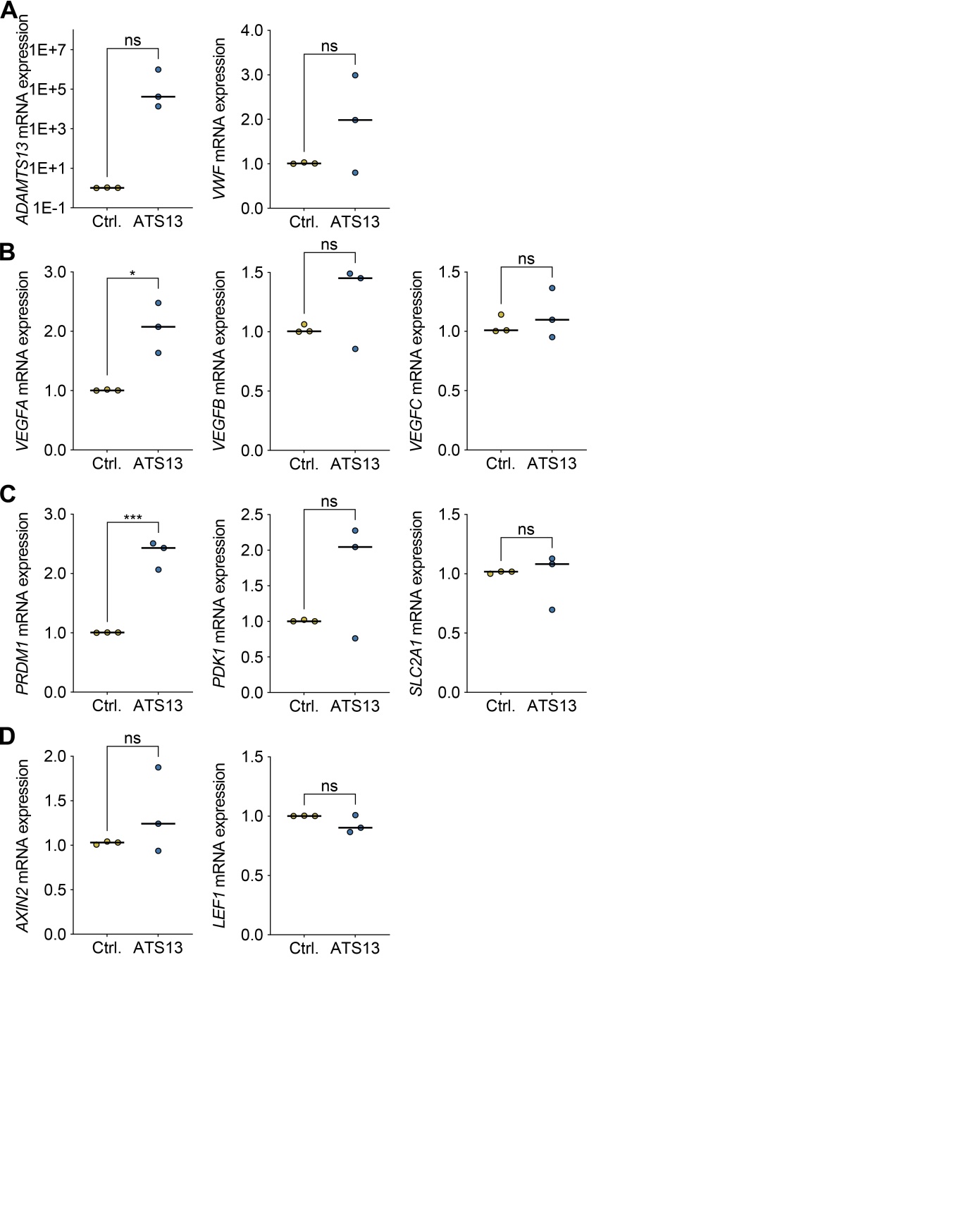


**FIGURE S15** Transient overexpression of ADAMTS13. Semiquantitative real-time PCR analysis for *ADAMTS13* (to confirm the overexpression) and its substrate *VWF* **(A)**, members of the VEGF family *VEGFA*, *VEGFB*, and *VEGFC* **(B)**, the hypoxia-responsive genes *PRDM1*, *PDK1*, and *SLC2A1* **(C)**, and Wnt target genes *AXIN2* and *LEF1* **(D)** 24 h after transfection of T3M4 cells with pcDNA3 (Ctrl., n=3) or pcDNA3-ADAMTS13 (ATS13, n=3). Each point represents the mean of two technical replicates. Expression was normalized to *RNA18S*. Mann-Whitney or t-test.

For ADAMTS13 overexpression, the coding sequence of wild-type ADAMTS13 was cloned into the overexpression vector pcDNA3. The cells were transfected with either the control vector pcDNA3 or the ADAMTS13 overexpression vector pcDNA3-ADAMTS13 using Lipofectamine3000 according to the manufacturer's instructions. The vector pcDNA3-ADAMTS13-EGFP served as a transfection control. 24 h after transfection, the medium was refreshed, and the cells were incubated for an additional 24 h before being harvested for analysis.


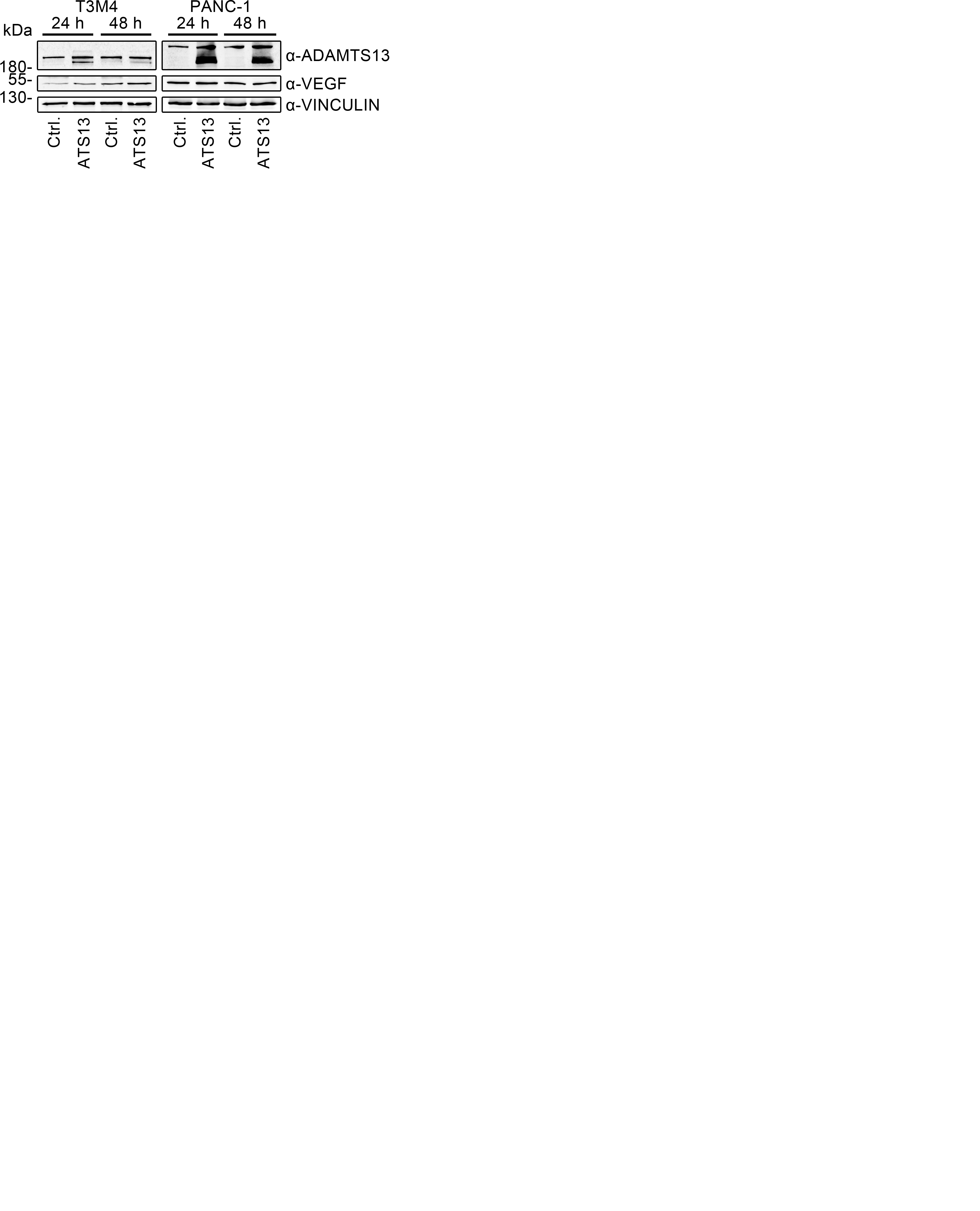


**FIGURE S16** Induction of VEGF upon transient overexpression of ADAMTS13. Immunoblot analysis of VEGF 48 h after transfection of PANC-1 or T3M4 cells with pcDNA3 (Ctrl.) or pcDNA3-ADAMTS13 (ATS13). Vinculin served as loading control. Molecular weight in kDa.


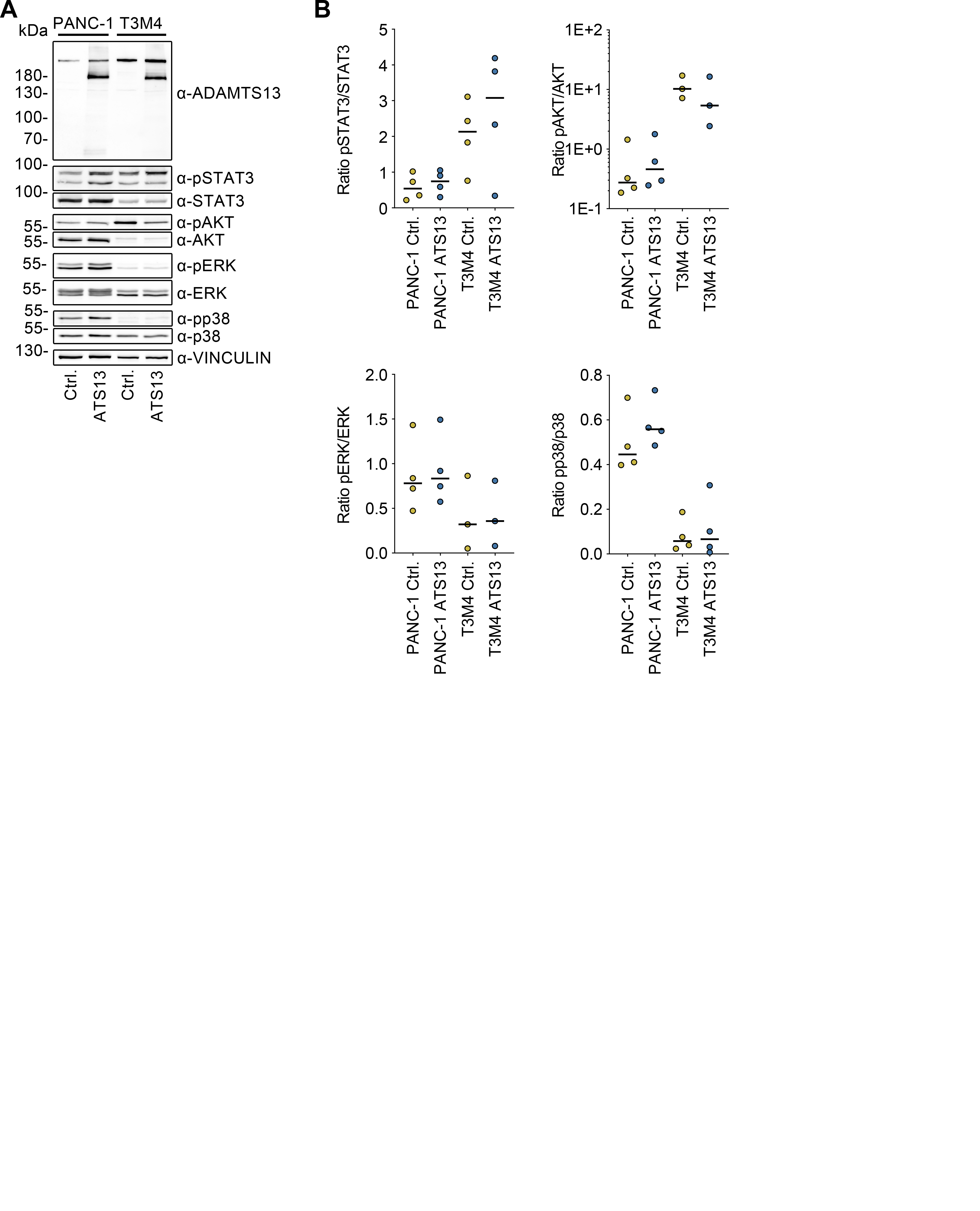


**FIGURE S17** Activation of STAT3-signaling upon transient overexpression of ADAMTS13. **(A)** Immunoblot analysis of ADAMTS13 as well as STAT3, phospho-STAT3 (pSTAT3), AKT, phospho-AKT (pAKT), ERK, phospho-ERK (pERK), p38, and phospho-p38 (pp38) 48 h after transfection of PANC-1 or T3M4 cells with pcDNA3 (Ctrl., n=3/4) or pcDNA3-ADAMTS13 (ATS13, n=3/4). Vinculin served as loading control. Molecular weight in kDa. **(B)** Quantification of all immunoblots performed (n=3/4).


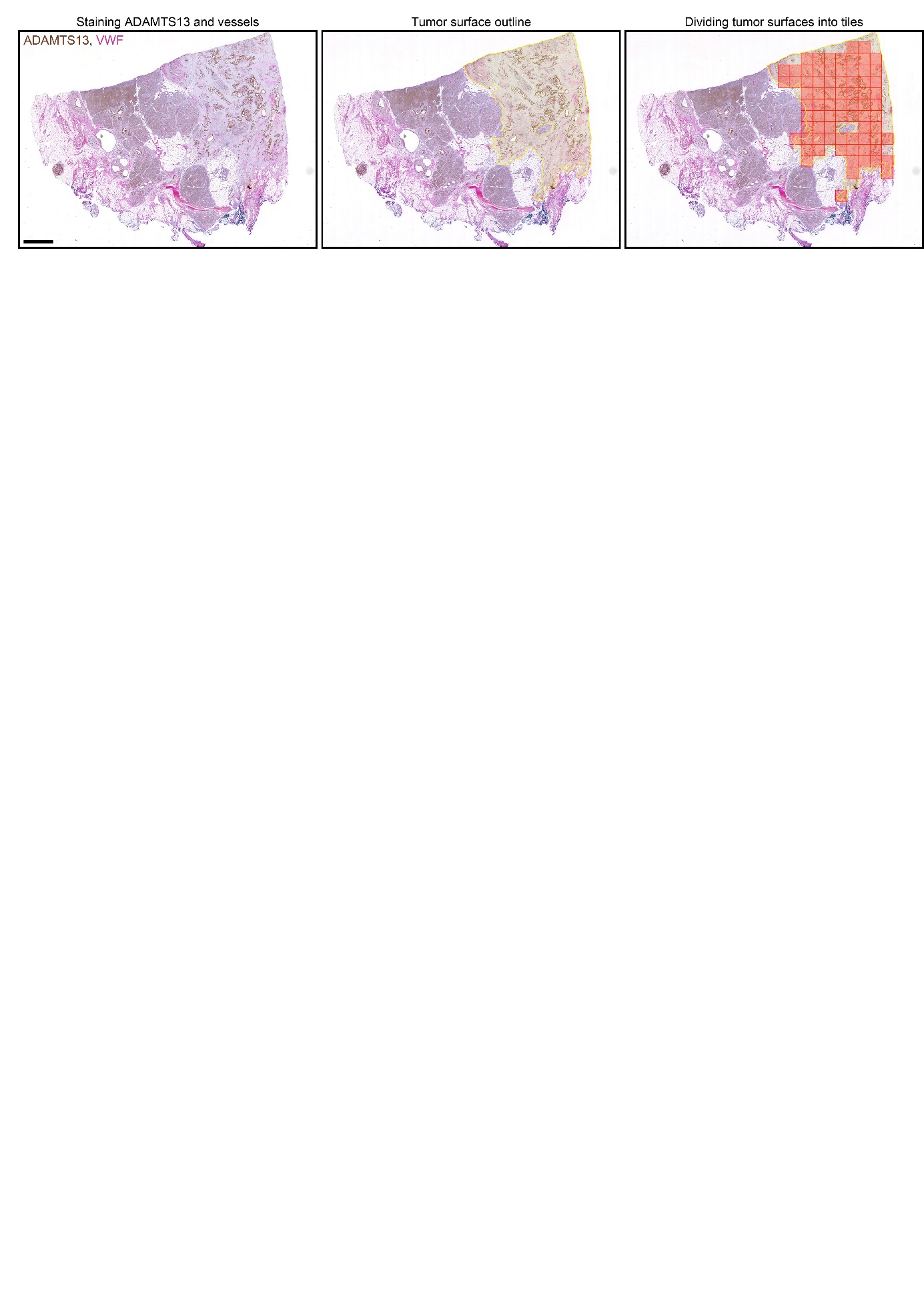


**FIGURE S18** Correlation between ADAMTS13 expression and tumor vascularization in PDAC whole tissue sections. Workflow: The tumor surface was outlined (yellow line), and the annotated area was divided into tiles of 800 x 800 pixels (red squares). QuPath was used to determine the DAP-positive pixels (ADAMTS13 staining) for each tile. Scale bar 2 mm.


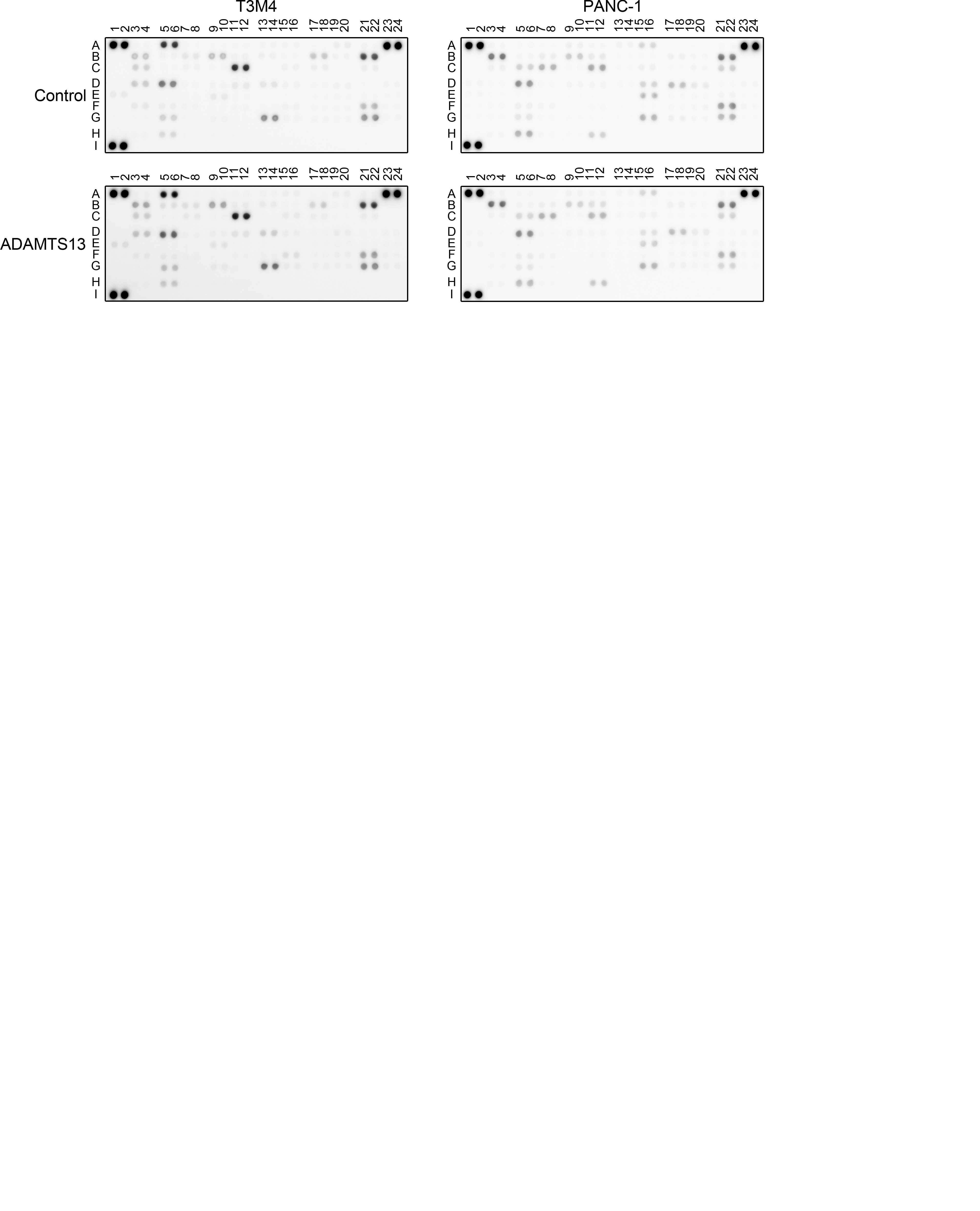


**FIGURE S19** Proteome Profiler Human XL Oncology Array. Immunoblot analysis of proteome profiler array for 84 cancer-related proteins (Proteome Profiler Human XL Oncology Array, R&D Systems) **(A)**. PANC-1 or T3M3 were transiently transfected with either pcDNA3 (Ctrl.) or pcDNA3-ADAMTS13 (ATS13), and proteins were isolated 48 h post-transfection.

**Table S1. Primary antibodies**

| **Antibody** | **Host, clonality** | **Application** |
| --- | --- | --- |
| ADAMTS13 (JG39-21), MA5-34796, Thermo Fisher Scientific | Rabbit, monoclonal | IHC: 1:500, 30 min, pH: 6.1; IB: 1:2000 |
| LEPR, LS-C332318, LSBio | Rabbit, polyclonal | IHC: 1:1000, 30 min, pH: 6.1 |
| MMP2 (D8N9Y), 13132, Cell Signaling, | Rabbit,  monoclonal | IHC:1:100, 60 min, pH: 6.1 |
| MMP9 (G657), 2270, Cell Signaling | Rabbit,  polyclonal | IHC:1:50, 60 min, pH: 6.1 |
| MMP12 (2G3), MA5-38368, Thermo Fisher Scientific | Rabbit, monoclonal | IHC: 1:500, 720 min, pH: 6.1; IB: 1:1000 |
| Serpin A12/Vaspin, (EPR23079-196), ab267470, Abcam | Rabbit, monoclonal | IB: 1:1000 |
| phospho-Histone H3 (Ser10), 06-570, MERCK; | Rabbit, polyclonal | IF: 1:300 |
| VWF, IR52761-2, Agilent | Rabbit, polyclonal | IHC: ready-to-use, 20 min or 120 min (human tissue or CAM, respectively), pH: 6.1 |
| Ki-67 (MIB-1), IR62661-2, Agilent | Mouse, monoclonal | IHC: ready-to-use, 35 min, pH: 6.1 |
| Leptin, sc-48408, Santa Cruz Biotechnology | Mouse, monoclonal | IHC: 1:500, 720 min, pH: 6.1 |
| Serpin A12/Vaspin, MAB4410, R&D Systems | Mouse, monoclonal | IHC: 1:50, 30 min, pH: 6.1 |
| VEGF (JH121, sc-57496, Santa Cruz Biotechnology | Mouse, monoclonal | IB: 1:500 |
| Vimentin (Clone V9),  IR630, Agilent, Dako | Mouse, monoclonal | IHC: ready-to-use, 35 min, pH: 6.1 |
| VINCULIN (hVIN-1), V9131, Sigma-Aldrich | Mouse, monoclonal | IB: 1:200 |
| β TUBULIN (TUB 2.1), sc-58886, Santa Cruz Biotechnology | Mouse, monoclonal | IB: 1:300 |

Primary Antibodies used in immunohistochemistry (IHC), immunoblotting (IB) and immunofluorescence (IF).

**Table S2. Oligonucleotides**

| **Gene** | **Species** | **Sequence (5’ > 3’)** |
| --- | --- | --- |
| *ADAMTS13* forward | Human | 5’-CAGGTTTACAGGCGGTATGG-3’ |
| *ADAMTS13* reverse | Human | 5’-CGTGGCTTAGGCTGGAAGTAG-3’ |
| *ADAMTS13* G1 forward | Human | 5’-CACCgGCTTCCAGAGGCAGAGGCAG-3’ |
| *ADAMTS13* G1 reverse | Human | 5’-AAACCTGCCTCTGCCTCTGGAAGCc-3’ |
| *ADAMTS13* G2 forward | Human | 5’-CACCgGGTGAGCCTGGAAGACATCG-3’ |
| *ADAMTS13* G2 reverse | Human | 5’-AAACCGATGTCTTCCAGGCTCACCc-3’ |
| *AXIN2* forward | Human | 5‘-CACGGAAACTGTTGACAGTGGATAC-3‘ |
| *AXIN2* reverse | Human | 5‘-GGTGGCTGGTGCAAAGACATAG-3‘ |
| *CDH1* forward | Human | 5‘-CTGGGCAGAGTGAATTTTG-3‘ |
| *CDH1* reverse | Human | 5‘-GACTGTAATCACACCATCTG-3‘ |
| *CDH2* forward | Human | 5‘-ACATATGTGATGACCGTAAC-3‘ |
| *CDH2* reverse | Human | 5‘-TTTTTCTCGATCAAGTCCAG-3‘ |
| *CST6* forward | Human | 5’-AAGACCAGGGTCACTGGAGA-3’ |
| *CST6* reverse | Human | 5’-CGGGGACTTATCACATCTGC-3’ |
| *DSP* forward | Human | 5‘-AGGCATCTAATAGGATTCAGG-3‘ |
| *DSP* reverse | Human | 5‘-CCTCTAGAGTTGATTTTGCAC-3‘ |
| *KLK13* forward | Human | 5’-GTGAATTACCCCAAAACTCTACAATGT-3’ |
| *KLK13* reverse | Human | 5’-AGACTTGACGACACTCCTCATCTG-3’ |
| *LEF1* forward | Human | 5‘-AGAGAGAGAAACTACAGGAATC-3‘ |
| *LEF1* reverse | Human | 5‘-CCACCATGTTTCAGATGTAG-3‘ |
| *MMP12* forward | Human | 5’-TGCTGATGACATACGTGGCA-3’ |
| *MMP12* reverse | Human | 5’-AGGATTTGGCAAGCGTTGG-3’ |
| *MMP2* forward | Human | 5‘-CTTCCAAGTCTGGAGCGATGT-3‘ |
| *MMP2* reverse | Human | 5‘-TACCGTCAAAGGGGTATCCAT-3‘ |
| *MMP9* forward | Human | 5‘-TTGACAGCGACAAGAAGTGG-3‘ |
| *MMP9* reverse | Human | 5‘-ACATAGGGTACATGAGCGCC-3‘ |
| *PDK1* forward | Human | 5‘-ATGATGTCATTCCCACAATG-3‘ |
| *PDK1* reverse | Human | 5‘-AAGAGTGCTGATTGAGTAAC-3‘ |
| *PRDM1* forward | Human | 5‘-TCCAGCACTGTGAGGTTTCA-3‘ |
| *PRDM1* reverse | Human | 5‘-TCAAACTCAGCCTCTGTCCA-3‘ |
| *RNA18S* forward | Human | 5’-CATGGCCGTTCTTAGTTGGT-3’ |
| *RNA18S* reverse | Human | 5’-ATGCCAGAGTCTCGTTCGTT-3’ |
| *SDC1* forward | Human | 5‘-TACTAATTTGCCCCCTGAAG-3‘ |
| *SDC1* reverse | Human | 5‘-GATATCTTGCAAAGCACCTG-3‘ |
| *SERPINA12* forward | Human | 5’-CGGTGAAAGGTCTTCTAAAGCC-3’ |
| *SERPINA12* reverse | Human | 5’-AGCCTAAGTCCATGTTCTGCC-3’ |
| *SLC2A1* forward | Human | 5‘-ACCTCAAATTTCATTGTGGG-3‘ |
| *SLC2A1* reverse | Human | 5‘-GAAGATGAAGAACAGAACCAG-3‘ |
| *SNAI1* forward | Human | 5‘-GAGTTTACCTTCCAGCAGCC-3‘ |
| *SNAI1* reverse | Human | 5‘-CAGAGTCCCAGATGAGCATT-3‘ |
| *SNAI2* forward | Human | 5‘-CAGTGATTATTTCCCCGTATC-3‘ |
| *SNAI2* reverse | Human | 5‘-CCCCAAAGATGAGGAGTATC-3‘ |
| *TJP1* forward | Human | 5‘-TTGTCTTCAAAAACTCCCAC-3‘ |
| *TJP1* reverse | Human | 5‘-GACTCACAGGAATAGCTTTAG-3‘ |
| *VEGFA* forward | Human | 5‘-AATGTGAATGCAGACCAAAG-3‘ |
| *VEGFA* reverse | Human | 5‘-GACTTATACCGGGATTTCTTG-3‘ |
| *VIM* forward | Human | 5‘-CAGATGCGTGAAATGGAAGAGAA-3‘ |
| *VIM* reverse | Human | 5‘-TAGGTGGCAATCTCAATGTCAA-3‘ |
| *ZEB1* forward | Human | 5‘-AAAGATGATGAATGCGAGTC-3‘ |
| *ZEB1* reverse | Human | 5‘-TCCATTTTCATCATGACCAC-3‘ |
| *ANGPT1* forward | Avian | 5‘-AGAAAACTCACGGATGTTG-3‘ |
| *ANGPT1* reverse | Avian | 5‘-TCTAGCTTGTAGGTTGACAG-3‘ |
| *CDH5* forward | Avian | 5‘-CTACCAACTCTGACTCAGGCTC-3‘ |
| *CDH5* reverse | Avian | 5‘-GCTGGAGCCAAGAACAATGAG-3‘ |
| *KDR* forward | Avian | 5‘-ATTGGACAGCATCACGAGCA-3‘ |
| *KDR* reverse | Avian | 5‘-GGTCCTCCATGGTCAAAGGG-3‘ |
| *PECAM1* forward | Avian | 5‘-GCAGACAGAGTTGTGTGGCA-3‘ |
| *PECAM1* reverse | Avian | 5‘-TGCCAAGAAGCTAATATGACATTGA-3‘ |
| *VWF* forward | Avian | 5‘-TTCCACGTAGAAGAAGATGCTCC-3‘ |
| *VWF* reverse | Avian | 5‘-AAAGGCTGCAACGGGAAACT-3‘ |

Primers employed for semi-quantitative real-time PCR and oligonucleotides for the cloning of guide RNAs to target exon 3 of *ADAMTS13* in a CRISPR/Cas-based manner.

**Table S3.** Patient data of the TMA used for immunohistochemistry in Figure S5 (n=41)

|  |  | **Number of patients**  **(total: n=41)** |
| --- | --- | --- |
| Gender (female:male) | 25:16 |  |
| Age (years) | 46-77 (mean: 66, median: 67) |  |
| Tumor size *ypT | ypT1 | 11 |
|  | ypT2 | 23 |
|  | ypT3 | 6 |
|  | ypT4 | 1 |
| Lymph node metastases *yN | ypN0 | 14 |
|  | ypN1 | 15 |
|  | ypN2 | 11 |
|  | ypNX | 1 |
| Distant metastases *yM | ypM0: no distant metastases | 32 |
|  | ypM1: distant metastases | 4 |
|  | No data | 5 |
| Histological grading | Not applicable | 41 |

**Table S4. Quantitation of the Proteome Profiler Human XL Oncology Array**

| **Coordinates** | **Protein** | **PANC-1**  **Ratio: ATS13/Ctrl.**  **[Fold-change]** | **T3M4**  **Ratio: ATS13/Ctrl.**  **[Fold-change]** |
| --- | --- | --- | --- |
| A3, A4 | α-Fetoprotein | 1.12 | 1.03 |
| A5, A6 | Amphiregulin | 1.00 | 1.11 |
| A7, A8 | Angiopoietin-1 | 0.69 | 1.41 |
| A9, A10 | Angiopoietin-like 4 | 0.63 | 1.21 |
| A11, A12 | ENPP-2/Autotaxin | 0.99 | 1.11 |
| A13, A14 | Axl | 0.53 | 1.02 |
| A15, A16 | BCL-x | 1.01 | 1.39 |
| A17, A18 | CA125/MUC16 | 0.78 | 1.17 |
| A19, A20 | E-Cadherin | 0.69 | 0.84 |
| A21, A22 | VE-Cadherin | 0.88 | 1.35 |
| B3, B4 | CapG | 1.11 | 1.43 |
| B5, B6 | Carbonic Anhydrase IX | 1.02 | 2.46 |
| B7, B8 | Cathepsin B | 0.64 | 1.04 |
| B9, B10 | Cathepsin D | 0.87 | 1.26 |
| B11, B12 | Cathepsin S | 1.47 | 1.33 |
| B13, B14 | CEACAM-5 | 1.02 | 1.17 |
| B15, B16 | Decorin | 0.65 | 0.46 |
| B17, B18 | Dkk-1 | 0.87 | 0.65 |
| B19, B20 | DLL1 | 0.75 | 0.79 |
| B21, B22 | EGF R/ErbB1 | 1.03 | 0.99 |
| C3, C4 | Endoglin/CD105 | 0.78 | 0.98 |
| C5, C6 | Endostatin | 0.95 | 0.93 |
| C7, C8 | Enolase 2 | 1.08 | 1.87 |
| C9, C10 | eNOS | 0.75 | 0.36 |
| C11, C12 | EpCAM/TROP1 | 0.83 | 1.02 |
| C13, C14 | ERα/NR3A1 | 1.23 | 2.52 |
| C15, C16 | ErbB2 | 0.89 | 1.25 |
| C17, C18 | ErbB3/Her3 | 0.86 | 0.64 |
| C19, C20 | ErbB4 | 0.38 | 2.19 |
| C21, C22 | FGF basic | 1.01 | 0.74 |
| D1, D2 | FoxC2 | 0.67 | 3.27 |
| D3, D4 | FoxO1/FKHR | 0.86 | 1.03 |
| D5, D6 | Galectin-3 | 1.07 | 1.06 |
| D7, D8 | GM-CSF | 1.17 | 0.95 |
| D9, D10 | CG α/β (HCG) | 1.00 | 1.02 |
| D11, D12 | HGF R/c-Met | 0.72 | 1.01 |
| D13, D14 | HIF-1α | 0.35 | 1.81 |
| D15, D16 | HNF-3β | 0.65 | 0.89 |
| D17, D18 | HO-1/HMOX1 | 0.94 | 1.01 |
| D19, D20 | ICAM-1/CD54 | 0.88 | 1.14 |
| D21, D22 | IL-2 Rα | 0.70 | 1.01 |
| D23, D24 | IL-6 | 0.86 | 0.81 |
| E1, E2 | CXCL8/IL-8 | 1.49 | 1.84 |
| E3, E4 | IL-18 BPa | 1.24 | 0.89 |
| E5, E6 | Kallikrein 3/PSA | 1.22 | 1.99 |
| E7, E8 | Kallikrein 5 | 1.12 | 1.10 |
| E9, E10 | Kallikrien 6 | 1.83 | 0.91 |
| E11, E12 | Leptin | 1.47 | 0.88 |
| E13, E14 | Lumican | 0.13 | 1.05 |
| E15, E16 | CCL2/MCP-1 | 0.75 | 0.40 |
| E17, E18 | CCL8/MCP-2 | 0.15 | 1.77 |
| E19, E20 | CCL7/MCP-3 | 0.80 | 1.13 |
| E21, E22 | M-CSF | 0.82 | 1.21 |
| E23, E24 | Mesothelin | 0.87 | 1.34 |
| F1, F2 | CCL3/MIP-1α | 1.09 | 5.38 |
| F3, F4 | CCL20/MIP-3α | 1.21 | 0.62 |
| F5, F6 | MMP-2 | 1.29 | 0.93 |
| F7, F8 | MMP-3 | 1.34 | 0.83 |
| F9, F10 | MMP-9 | 0.88 | 1.68 |
| F11, F12 | MSP/MST1 | 0.96 | 1.23 |
| F13, F14 | MUC-1 | 0.96 | 2.18 |
| F15, F16 | Nectin-4 | 1.91 | 2.84 |
| F17, F18 | Osteopontin (OPN) | 1.12 | 0.93 |
| F19, F20 | p27/Kip1 | 0.99 | 6.00 |
| F21, F22 | p53 | 0.71 | 1.33 |
| F23, F24 | PDGF-AA | 0.75 | 0.91 |
| G1, G2 | CD31/PECAM-1 | 1.15 | 1.89 |
| G3, G4 | Progesterone R/NR3C3 | 1.14 | 1.41 |
| G5, G6 | Progranulin | 0.87 | 1.25 |
| G7, G8 | Prolactin | 1.54 | 1.15 |
| G9, G10 | Prostasin/Prss8 | 0.92 | 1.89 |
| G11, G12 | E-Selectin/CD62E | 1.06 | 2.24 |
| G13, G14 | Serpin B5/Maspin | 1.07 | 1.22 |
| G15, G16 | Serpin E1/PAI-1 | 1.06 | 1.81 |
| G17, G18 | Snail | 0.85 | 1.20 |
| G19, G20 | SPARC | 0.91 | 2.43 |
| G21, G22 | Survivin | 0.53 | 1.00 |
| G23, G24 | Tenascin C | 0.89 | 1.70 |
| H1, H2 | Thrombospondin-1 | 0.77 | 1.10 |
| H3, H4 | Tie-2 | 1.21 | 1.59 |
| H5, H6 | u-Plasminogen Activator/Urokinase | 0.88 | 1.28 |
| H7, H8 | VCAM-1/CD106 | 0.97 | 1.51 |
| H9, H10 | VEGF | 0.99 | 1.58 |
| H11, H12 | Vimentin | 1.20 | 1.29 |

Quantification of the Proteome Profiler Human XL Oncology Array (Figure S16). The values presented herein represent the ratio of quantified band intensity from the overexpression group (transfection with pcDNA3-ADAMTS13) to the control group (transfection with pcDNA3). The first column shows the position of the corresponding factors on the array.

**References**

1. Bankhead P, Loughrey MB, Fernandez JA, et al. QuPath: Open source software for digital pathology image analysis. *Sci Rep*. 2017;7(1):16878.

2. Cancer Genome Atlas Research Network. Electronic address aadhe, Cancer Genome Atlas Research N. Integrated Genomic Characterization of Pancreatic Ductal Adenocarcinoma. *Cancer Cell*. 2017;32(2):185-203 e13.

3. Schneider CA, Rasband WS, Eliceiri KW. NIH Image to ImageJ: 25 years of image analysis. *Nat Methods*. 2012;9(7):671-5.
